# HDAC6 orchestrates metastatic and immunosuppressive programs in small cell lung cancer through S100A2-TGF-β/SMAD and CSF1R signaling

**DOI:** 10.1186/s12943-025-02552-y

**Published:** 2026-01-07

**Authors:** Yantao Jiang, Junjie Yu, Ting Wang, Qingwu Du, Jingya Wang, Yi Lu, Qi Xu, Huiyan Liu, Xueyang Li, Luyao Tong, Tingting Qin, Dingzhi Huang

**Affiliations:** 1 Tianjin Medical University Cancer Institute and Hospital, National Clinical Research Center for Cancer, Key Laboratory of Cancer Prevention and Therapy, Tianjin, P. R. China; 2 Tianjin’s Clinical Research Center for Cancer, Department of Thoracic Oncology, Tianjin Lung Cancer Center, Tianjin Cancer Institute & Hospital, Tianjin Medical University, Huanhuxi Road, Hexi District, Tianjin, 300060 P. R. China

**Keywords:** HDAC6, S100A2, SCLC, EMT, CSF1R

## Abstract

**Background:**

Small cell lung cancer (SCLC) remains a highly lethal malignancy with limited therapeutic options. The purpose of this study was to investigate the central role of histone deacetylase 6 (HDAC6) in SCLC progression and its regulatory mechanisms to identify novel therapeutic strategies.

**Methods:**

Preclinical SCLC models were utilized alongside molecular, cellular, and immunological techniques to elucidate HDAC6's mechanistic functions. The deacetylation of S100A2 and its impact on downstream signaling were analyzed, compensatory responses to HDAC6 inhibition were assessed, and the efficacy of dual-target inhibition was evaluated.

**Results:**

HDAC6 was found to deacetylate the calcium-binding protein S100A2 at lysine 27, thereby stabilizing TGF-β/SMAD signaling to promote epithelial-mesenchymal transition (EMT) and metastatic dissemination. Simultaneously, HDAC6 polarized macrophages toward tumor-promoting M2 phenotypes, fostering an immunosuppressive microenvironment. HDAC6 inhibition triggered compensatory CSF1R upregulation, revealing a resistance mechanism. Dual blockade of HDAC6 and CSF1R synergistically suppressed primary tumor growth and metastasis while reprogramming macrophages toward anti-tumor M1 states. SCLC patients with co-high expression of HDAC6 and CSF1R exhibited worse progression-free survival (PFS).

**Conclusion:**

This study defines the HDAC6-S100A2-TGF-β/SMAD and HDAC6-CSF1R-macrophage axes as actionable therapeutic vulnerabilities. The dual inhibition strategy provides a translational framework to overcome stromal and immune barriers in this recalcitrant cancer.

**Supplementary Information:**

The online version contains supplementary material available at 10.1186/s12943-025-02552-y.

## Introduction

Small cell lung cancer (SCLC) is a highly aggressive neuroendocrine carcinoma with poor prognosis, accounting for approximately 15% of all lung cancer cases [1]. Platinum-etoposide chemotherapy as remains the first-line standard of care [2, 3], and the recent incorporation of immune checkpoint inhibitors (ICIs) has yielded modestly improved outcomes [4]. However, patients with extensive-stage SCLC (ES-SCLC) still demonstrates a median overall survival (mOS) of only 12–13 months, with 60% of patients experiencing disease recurrence within 3 months [2]. Unlike non-small cell lung cancer (NSCLC), SCLC lacks common druggable oncogenic mutations and is primarily characterized by biallelic inactivation of tumor suppressor genes TP53 and RB1, MYC family amplification, and dysregulated epigenetic modifications including aberrant histone methylation and acetylation [5, 6]. Molecular profiling has enabled the classification of SCLC into four transcriptional subtypes based on differential expression of ASCL1, NEUROD1, POU2F3, and YAP1 (ANPY signature) [7]. However, this classification framework has not translated into meaningful targeted therapeutic strategies. The identification of novel molecular targets and the elucidation of their mechanisms remain urgent priorities for the development of precision therapies for this lethal malignancy.

SCLC is characterized by early dissemination, with ES-SCLC accounting for 70%, and up to 15% of patients already harboring brain metastases [2, 8]. Although initially sensitive to chemotherapy, most patients relapse within 6 months, underscoring the clinical challenge posed by rapid disease recurrence [9]. The high metastatic propensity and chemoresistance of SCLC highlight the need to dissect the underlying biological mechanisms. Recent work has implicated cellular plasticity programs in driving metastasis, immune evasion, and therapeutic failure in SCLC. The study by Joseph M. Chan et al. revealed that PLCG2 promotes tumor progression in SCLC by driving pro-metastatic and stem-like phenotypes, while its high expression is associated with an immunosuppressive microenvironment, collectively accelerating malignant metastasis and leading to poor prognosis [10]. Among the emerging themes, epigenetic regulation has attracted considerable interest as a central determinant of SCLC progression and therapy resistance. Cancer cells can evade chemotherapy through epigenetic modifications, particularly aberrant histones or genomic DNA modifications [11]. Histone acetylation is a pivotal epigenetic modification that governs chromatin accessibility and transcriptional programs in cancer [12, 13]. This sophisticated process is dynamically regulated by histone acetyltransferases (HATs) and histone deacetylases (HDACs), which dynamically regulate gene transcription by respectively adding or removing acetyl groups from histone proteins, thereby inducing chromatin remodeling [14]. HDACs are primarily classified into two major categories: zinc-dependent HDACs (classes I, II, and IV) and NAD^+^-dependent sirtuins (class III), comprising 18 distinct subtypes in total [15]. In SCLC, aberrant epigenetic regulation—including evasion of innate immunity—has been implicated in tumor progression and metastasis. A study revealed that pan-HDAC inhibitors reversed NKG2DL silencing in SCLC, restoring innate immune recognition while suppressing neuroendocrine differentiation—bridging immune activation and lineage reprogramming [16]. Our previous studies identified a unique SCLC subtype with elevated HDAC7 expression [7]. We demonstrated that HDAC7 directly upregulates XPO1 and MYC expression, functioning as an oncoprotein in SCLC. Patients with this HDAC7-positive subtype exhibited significantly poorer survival outcomes. These findings position XPO1 as a highly promising therapeutic target in HDAC7-mediated SCLC pathogenesis. The roles of other HDAC family members in SCLC metastasis remain poorly understood.

HDAC6 is a unique member of HDAC family, distinguished by being the sole isoform containing two catalytic domains (CDs) and a zinc-finger ubiquitin-binding domain (Zf-UBD) [15, 17]. Unlike most HDACs, HDAC6 exhibits broad substrate specificity, mediating deacetylation not only of histones but also diverse non-histone proteins including HSP90 and α-tubulin, thereby regulating chaperone activity and microtubule dynamics [13, 18]. Multiple studies have demonstrated that HDAC6 OE functions as an oncogenic driver across various malignancies, including colorectal cancer [19], lung cancer [20], breast cancer [21], and renal cell carcinoma [22], where it promotes tumor progression and correlates with poor clinical outcomes. Building upon our earlier findings that HDAC6 OE drives epithelial-mesenchymal transition (EMT) in NSCLC through PI3K/AKT/mTOR pathway activation, thereby augmenting tumor proliferation and invasive capacity [12]. A preclinical study has shown that co-targeting HDAC6 with ACY-1215 and BET proteins with JQ1 synergistically enhances NK cell-dependent antitumor immunity in SCLC models [6]. However, HDAC6's multifunctional roles depend on its formation of protein complexes, and whether it can serve as a screening target for novel subtypes in SCLC along with its specific functional substrates remains unknown.

In this study, we identified HDAC6 overexpression as both a prognostic risk factor and a molecular signature defining a distinct SCLC subtype. Mechanistically, we uncover S100A2 as a novel non-histone substrate of HDAC6, which directly deacetylates S100A2 at lysine 27 (K27) to enhance its activity. As an EF-hand calcium-binding protein of the S100 family, S100A2 interacts with cytoskeletal proteins and cyclins to regulate multiple signaling pathways. Our findings demonstrate that S100A2 acetylation status modulates its binding affinity for SMAD3 and the phosphorylation stability of SMAD3 at Ser423/425 and SMAD2 at Ser465/467, ultimately affecting the stability of SMAD2/3/4 complex formation, nuclear translocation, and subsequent transcriptional activation of SNAI2-collectively governing EMT-mediated malignant progression in SCLC. In parallel, we revealed HDAC6-mediated deacetylation of CSF1R at H3K27, with HDAC6 knockout upregulating CSF1R expression, suggesting CSF1R's potential role in therapeutic resistance. Based on compelling evidence from xenograft models and patient-derived samples, we propose that co-targeting HDAC6 (Tubastatin A) and CSF1R may represent a promising therapeutic strategy for patients with HDAC6-high SCLC.

## Methods

### Ethics statement, patients and tissues

In our clinical dataset, 98 SCLC samples that underwent surgery between January 2012 and December 2022 were obtained from Tianjin Cancer Institute and Hospital, which was approved by the ethics committee of the Tianjin Medical University Cancer Institute and Hospital and adhered to the ethical guidelines of the Helsinki Declaration. All patients provided written consent for the use of their specimens and data. Patients who were assessed as eligible for surgery, had not received any prior treatment before the operation, and had complete follow-up information were included in this study. All patients were identified as either male or female in terms of gender. Termination time of follow-up of our clinical data was May 20, 2024. In all, 6 pairs SCLC tumor tissue and the corresponding normal tissue samples for protein extraction were freshly frozen in dry ice and stored at − 80 °C until used. The animal experiment was conducted under an approved from the Animal Care and Use Committee of Tianjin Cancer Institute & Hospital of Tianjin Medical University.

### Samples, cell lines and cell culture

The expression of HDAC family in the four subtypes of SCLC and the relationship with clinical staging were downloaded from the George et al. cohort [23]. HDAC6 and S100A2 gene expression were downloaded from GEO datasets (https://www.ncbi.nlm.nih.gov/) and TCGA database (https://portal.gdc.cancer.gov/). Survival map data on the relationship between S100A2 gene expression and patient survival were obtained from the GEPIA2.0 database ((http://gepia.cancer-pku.cn/).

The human SCLC cell lines (SBC-2, NCI-H1688, NCI-H146, NCI-H446, DMS53, NCI-H196, SW1271, NCI-H526) were cultured in RPMI 1640 medium (Gibco, USA) with 10% fetal bovine serum (Zeta life, France) and penicillin–streptomycin solution (50 µg/mL). Non-SMC, HEK293T cell, BEAS-2B cell, HBMEC and NHA cells were cultured in DMEM medium (Gibco, USA), with all other conditions being the same as for other cells. All cells were authenticated with STR profiling and were maintained at 37 °C under humidified atmosphere containing 5% CO2. The mouse SCLC cell line (non-SMC) was a generous gift from Professor Hongbin Ji at the Center for Excellence in Molecular Cell Science at the Chinese Academy of Sciences. Other cell lines used in this study were purchased from American Type Culture Collection (ATCC) cell bank.

### Constructs and generation of overexpression (OE) or knockout (KO) cell lines

Lentivirus infection of SBC-2, NCI‐H1688 and HEK293T cells were conducted according to the protocol described in our previous publication [7, 12]. For the cell lines with stable knockout, sgRNA sequences (see Supplementary Table 2) targeting HDAC6 and SMAD3 were obtained from the sgRNA design tool (http://sam.genome-engineering.org/database/, Cas9-Activators with SAM) and cloned into lentiCRISPRv2. For the cell lines with overexpressed targeted genes, HDAC6 gene was cloned into pLenti-CMV-luc2-EGFP-Neo vectors accompanied by synonymous mutations in the GAAAGGACACGCAGCGATCT region, and S100A2 gene was cloned into pLenti-CMV-GFP-Hygro vectors. Target cells were transfected with lentiviral particles followed by Puromycin selection (2 μg/mL), Neomycin selection (1 mg/mL) and Hygromycin selection (500 μg/mL) according to the corresponding vectors, respectively.

Of these three stable cell lines, the most efficient one was used for the relevant assays.

### Western blotting (WB)

The protein extraction and western blotting processes were performed as described in our previous publication [24, 25]. The nuclear and cytoplasmic fractions were extracted by the NE-PER Nuclear and Cytoplasmic Extraction Reagents (#78,833, Thermo Scientific) according to manufacturer's protocol. The primary antibodies were listed below: GAPDH (1:1000 dilution, CST Cat#3700), HDAC6 (1:1000 dilution, Proteintech Cat#12,834–1-AP), N-cadherin (1:500 dilution, Santacruz Cat#sc-59987), E-cadherin (1:500 dilution, Santacruz Cat#sc-8426), Vimentin (1:500 dilution, Santacruz Cat#sc-6260), Snail (1:500 dilution, Santacruz Cat#sc-271977), Slug (1:500 dilution, Santacruz Cat#sc-166476), Bcl2 (1:1000 dilution, CST Cat#2764), Bax (1:1000 dilution, CST Cat#2772), Bim (1:1000 dilution, CST Cat#2933), Cleaved-casepase3 (1:1000 dilution, CST Cat#9664), S100A2 (1:1000 dilution, Abcam Cat#ab109494), acetylated α-Tubulin (1:500 dilution, Santacruz Cat#sc-23950), Ac-lysine (1:500 dilution, Santacruz Cat#sc-32268), FLAG (1:50,000 dilution, Proteintech Cat#20,543–1-AP), HA (1:5000 dilution, Proteintech Cat#51,064–2-AP), SMAD3 (1:5000 dilution, Proteintech Cat#66,516–1-Ig), p-SMAD3(Ser423/Ser425) (1:1000 dilution, CST Cat#9520), SMAD2 (1:5000 dilution, Proteintech Cat#12,570–1-AP), p-SMAD2(Ser465/Ser467) (1:1000 dilution, CST Cat#18,338), SMAD4 (1:1000 dilution, Proteintech Cat#10,231–1-AP), ZO-1 (1:1000 dilution, CST Cat#13,663), VE-cadherin (1:1000 dilution, CST Cat#2500), β-catenin (1:1000 dilution, CST Cat#8480), Claudin-5 (1:1000 dilution, Thermo Fisher Cat#35–2500), Histone 3 (1:2000 dilution, Proteintech Cat#17,168–1-AP).

### Gene expression analysis by real‑time quantitative PCR (qRT‑PCR)

The total RNA was extracted using the Trizol reagent following the product protocol (Invitrogen, No.15596026) and reverse transcribed total RNA into complementary DNA using PrimeScript™ RT reagent Kit (#RR420A, Takara, China). Subsequently, Universal Blue qPCR SYBR green Master Mix (Yeasen, China) was used for RT-qPCR. Primers used to analyze mRNA levels were listed in Supplementary Table 3.

### Immunohistochemistry (IHC)

IHC was used to examine the expression of HDAC6, CSF1R, CD86, CD206 and Ki67 in SCLC tissues. Antigen retrieval was performed with pressure cooking in tris–EDTA (pH = 9.0). The slides were incubated with primary antibodies at 4 ℃ overnight, followed by secondary antibodies at room temperature for 30 min. Chromogenic reactions were performed using a DAB kit (ZL1-9019; ZSGB-BIO, China). The expression of HDAC6, CSF1R, CD86, CD206 and Ki67 were evaluated using an H-score system. The H-score, ranging from 0 to 300, was calculated based on the ratio of the weighted sum of positive cells to the total number of detected cells, taking into account the staining intensity. The staining intensity was assessed using the following criteria: 0 for negative, 1 for weak positive, 2 for moderate positive, and 3 for strong positive. Three independent clinical pathologists, blinded to all patient information, evaluated all IHC results. We used antibody against HDAC6 (Proteintech, Cat#12,834–1-AP) at a dilution of 1:200, Ki67 (Proteintech, Cat#27,309–1-AP) at a dilution of 1:2000, CSF1R (Abways, Cat#AY0252) at a dilution of 1:50, CD86 (Abcam, Cat#ab220188) at a dilution of 1:100, CD206 (Reactivity: Mouse) (Proteintech, Cat#32,647–1-AP) at a dilution of 1:200 and CD206 (Reactivity: Human) (Abways, Cat#BY9003) at a dilution of 1:100.

### Immunofluorescent (IF)

Cells grown on glass coverslips were washed three times with PBS, fixed with 4% paraformaldehyde for 10 min, and permeabilized with PBS containing 0.5% Triton X-100 for 10 min. Cells were then blocked with 1% BSA for 1 h, followed by incubating with the indicated antibody overnight at 4 °C. Cells were incubated with goat anti-rabbit or mouse secondary antibodies (Alexa Flour 647 or 488, dilution 1:200) for 1 h at room temperature. The cells were treated with 1:1000 1 mg/mL DAPI for 5 min and then mounted by Antifading mounting medium. Cells were probed with the following primary antibodies: anti-HDAC6 antibody (Proteintech Cat#12,834–1-AP, 1:100 dilution), anti-S100A2 antibody (Abcam Cat#ab109494, 1:100 dilution), anti-MMP9 antibody (Santacruz Cat#sc-21733, 1:100 dilution), anti-E-cadherin antibody (Santacruz Cat#sc-8426, 1:100 dilution), anti-p-SMAD2 (Ser465/Ser467) antibody (1:400 dilution, CST Cat#18,338) and anti-SMAD4 antibody (Proteintech Cat#10,231–1-AP, 1:500 dilution).

### Cell proliferation

SBC-2 and NCI-H1688 cells infected with control lentivirus and HDAC6 KO lentivirus were seeded into 96-well plates at a density of 2000 cells per well. At 24, 48, 72 h, 96 h after the cells were seeded, CCK-8 reagent (Zeta, France) was mixed with the cells for 2 h incubation at 37 ℃ in the dark. The absorbance value was measured at 450 nm with Microplate reader.

Additionally, the plate colony formation assay was performed to evaluate the colony formation ability of tumor cells. The stably transfected cells were seeded in six-well plates, which was assessed with crystal violet staining after 14 days cell culture.

### Transwell assay

The transwell assay for cell migration or invasion was conducted using a 24-well culture insert. 3 × 10^4^ cells were suspended in 200 μl of serum-free RPMI-1640 media. The cell suspension was added to the upper chamber of the transwell insert, which was coated with or without a matrix component like Matrigel (BD Biosciences, NJ, USA) for invasion or migration assays. 600 μl of RPMI-1640 supplemented with 20% FBS was added to the lower.

chamber of the insert as a chemoattractant. The transwell chambers were incubated for 24 h to allow cells to migrate or invade through the porous membrane. After the incubation period, cells that did not migrate or invade through the membrane and remained on the upper surface of the chamber were carefully cleaned by gently wiping the surface with a cotton swab. The cells that passed through the membrane and reached the lower surface of the chamber were fixed in 4% paraformaldehyde (Solarbio, Beijing, China) and stained with crystal violet.

### Wound healing assay

The cells were seeded into six-well plates until cells confluence was 90%. The wound was induced by scraping the cell monolayer with a 100 μl pipette tip and left for 24 h or 48 h before photographing.

### Cell co-culture

To establish a coculture system for macrophages and SCLC cells in vitro, a 24 mm Transwell chamber with an 8 μm pore polycarbonate membrane (Corning, USA) was used. THP-1 monocytes (1 × 10^6^/well) were first induced to differentiate into M0 macrophages with 150 nM PMA (Sigma, USA) in the lower chamber. SBC-2 and NCI-H1688 cells (5 × 10^5^/well) were added to the upper chamber and co-cultured with M0 macrophages for 48 h. Total RNA was extracted from the cells in the lower chamber of each group, and the expression of typical M1 markers, such as CD86, and typical M2 markers, such as CD206, were detected by qRT-PCR.

### Macrophage migration assay

Firstly, conditioned media from different treatment groups of SBC-2 and NCI-H1688 cells need to be collected and placed in the lower chamber. 5 × 10^4^ THP-1 cells/200ul were placed into the upper chamber after PMA induced cell adherent growth. The rest of the steps were the same as transwell assay. The difference in macrophage migration between the groups was observed after 24 h.

### Construction of a Blood–Brain Barrier Model (BBB)

HBMECs and NHAs were co-cultured on opposite sides of a 24-well transwell polycarbonate insert (3 µm pore size, Coring) to develop the in vitro BBB model. The transwell insert was coated with 5ug/cm^2^ Collagen, Type I (Yeasen, Cat#40125ES10) for 1 h and placed upside-down. Then, 1 × 10^5^ NHAs were plated on the underside of the insert. The cells were incubating at 37℃ in 5% CO2 and being fed with astrocyte medium every 15–30 min. After 4 h, the inserts were turned over and placed into 24-well plates. Astrocyte medium (1 mL) was added to the lower chamber, and NHAs were incubated for an additional 24 h. Further, the upper chamber of the inserts was seeded with 1 × 10^5^ HBMECs and incubated for 3 d. The BBB barrier function assay was started 2–3 days after HBMEC cell inoculation using 4 h leakage assay, sodium fluorescein permeation assay, and expression of model tight junction proteins at different co-culture times. Combining the results of the above functional experiments, the time node with the strongest BBB barrier function was selected for the subsequent experiments. After the BBB construction was completed, 1 ml of 1 × 10^5^ cells with EGFP fluorescence of different treatments were added to the upper chamber, and the lower chamber was washed with PBS after 24 h and fixed with 4% paraformaldehyde for 20 min at room temperature, and finally observed under a confocal microscope. Five fields of view were randomly taken to count the relative migrated cell volume. P_Flu_% = (C_Flu_ lower chamber × V_Flu_ lower chamber)/(C_Flu_ upper chamber × V_Flu_ upper chamber) × 100%.

### In vivo tumor xenograft assays

NCI-H1688 wild-type cells and HDAC6 KO NCI-H1688 cells (1 × 10^7^/mouse in 0.1 mL with 50% Matrigel and 50% saline) were subcutaneously injected into the groins of 6-week-old female BALB/c nude mice (purchased from Jiangsu Gempharmatech, 5 mice/group). The body weight and tumor size of the mice were measured every three days, and the tumor burden in the two groups of mice was observed after one month. NCI-H1688 wild-type cells and HDAC6 KO NCI-H1688 cells (3 × 10^6^/mouse in 0.1 mL PBS) with luciferase plasmid were injected into mice via tail vein. Two weeks after injection, lung Computed Tomography (CT) and In Vivo Imaging System (IVIS) were performed every 1–2 weeks to monitor the tumor metastatic burden in both groups of mice. Mice were euthanized when the tumor metastatic burden was high or when mice exhibited > 20% weight loss, distress, or impaired mobility. After reaching the endpoint of the study, the lungs of the mice were dissected out and sectioned by paraffin embedding and stained with HE staining, and the lung metastases of the two groups of mice were observed.

### In vivo brain orthotopic xenograft assays

To establish the brain orthotopic xenograft model, 6-week-old female BALB/C nude mice were used. The mice were first anesthetized, and then positioned using a stereotactic instrument for small animals. The injection site was targeted at the striatum region of the right cerebral hemisphere, with the stereotactic coordinates set at X = + 0.8 mm, Y = + 1.8 mm, and Z = −3.2 mm relative to the bregma. A small burr hole approximately 1 mm in diameter was drilled at the determined injection site using a micro-drill. Subsequently, a micro-syringe was used to slowly inject 1 × 10⁶ NCI-H1688 cells (either HDAC6 WT or KO), stably expressing luciferase (luc), resuspended in 5 μL of PBS, into the mouse brain. The postoperative status of the mice in both groups was monitored, and body weight was measured every three days. The frequency of IVIS imaging and the study endpoint were consistent with the methods described previously.

### Intracardiac injection for establishing an in vivo metastasis model

A total of 2 × 10⁶ NCI-H1688 cells (HDAC6 WT or KO) stably expressing luc were resuspended in 100 μL of PBS and slowly injected into the left cardiac ventricle of 6-week-old female BALB/c-nude mice using a 26G needle. Prior to injection, the mice were anesthetized via gas inhalation. Successful injection was confirmed by the visible backflow of arterial blood into the syringe. The monitoring of metastatic burden and the study endpoint were consistent with the methods described previously.

### In vivo drug efficacy and side effects assay

5 × 10^6^ non-SMCs were resuspended in 100µL PBS and injected subcutaneously into BALB/c nude mice. And non-SMCs (1 × 10^6^/mouse in 0.1 mL PBS) with luciferase plasmid were injected into mice via tail vein. Mice which bear tumor (average size > 100 mm^3^) were randomly separated into four groups (6 mice/group) and treated with either vehicle control or Tubastatin A alone (MCE, 10 mg/kg, i.p., five times a week for two consecutive weeks.), BLZ945 (MCE, 50 mg/kg, i.g., five times a week for two consecutive weeks.) alone or their combination. Tumor size and metastatic burden were monitored as described previously. After reaching the endpoint of the study, lung, liver, and kidney tissues from each group of mice were taken for HE staining to observe the metastatic burden and toxic effects of the drugs. For immune profiling, enzymatically dissociated tumor single-cell suspensions (collagenase IV/DNase I, 2 h digestion) were prepared. Cells were stained with Zombie NIR™ viability dye (423,105, BioLegend) and antibody panels (F4/80, PerCP/Cyanine5.5, BioLegend; CD206, APC, BioLegend; CD86, PE, BioLegend), followed by acquisition on CytExpert software (Beckman Coulter, Brea, USA). Data analysis was performed using FlowJo v10 software (TreeStar Inc., USA). The animal experiment was conducted under an approved from the Animal Care and Use Committee of Tianjin Cancer Institute & Hospital of Tianjin Medical University.

### RNA sequencing

Total RNA was extracted from NCI-H1688 HDAC6 wide-type or NCI-H1688 HDAC6 KO cells using Trizol reagent (QIAGEN, USA). All samples were sent to BGI (Wuhan, China) for further RNA sequencing and analysis via Illumina HiSeq 2500 (San Francisco, CA, USA). The expression level of each gene was calculated as the fragments per kilobase of transcript per million mapped reads (FPKM) value. Differentially expressed genes (DEGs) between groups were identified by the edgeR package (http://www. rproject.org/) with thresholds of false discovery rate < 0.05 and absolute log2-fold change ≥ 2.

### CUT&TAG analysis

The CUT&TAG experiments were performed by Wuhan Zhenyue Bioinformatics Co., Ltd. Quality control and trimming of the reads were done using FastQC (v0.11.9, RRID:SCR_014583) Clean reads were aligned to the human genome (hg38) using Bowtie2(v2.4.5). PCR duplicates were removed using Picard MarkDuplicates. Peak calling was performed using Macs2. Peaks were filtered at a q-value threshold < 0.05. Peak calls from each replicate across both controls and treatments were merged into a union set using bedtools merge. Differential peaks were identified using DESeq2(v1.32.0, RRID:SCR_000154) with default parameters. Regions with a P‐value threshold < 0.05 (Log2FoldChange > 0.5 or < − 0.5) were considered to be statistically significant. Heatmaps were produced using ComplexHeatmap. Peaks were annotated to the nearest feature using ChIPseeker (v1.32.0). KEGG and GO analysis was performed using clusterProfiler (version 4.2.2, RRID:SCR_016884).

### Mass spectrometry-based label-free quantitative acetylated proteomics

Label-free quantitative acetylated proteomics was conducted by Beijing Genomics institution (Shenzhen, China). Briefly, proteins were extracted using lysis buffer (8 M urea, 100 mM Tris–HCl, pH 8.5) and digested with trypsin. Acetylated peptides were enriched using anti-acetyllysine antibody beads (BGI), then analyzed by LC–MS/MS on a Bruker timsTOF Pro system in DDA or DIA mode with 4D ion mobility separation. Raw data were processed using MaxQuant against the UniProt database for identification and label-free quantitation.

### Immunoprecipitation analysis

Immunoprecipitation assay was performed using the Pierce Classic Magnetic IP/Co-IP Kit (Thermo Scientific Pierce) according to manufacturer's instruction. Briefly, 1 mg of SCLC cells determined by the Pierce BCA protein assay. Then the supernatants were incubated with of bait antibody and 25 µL of Pierce protein A/G Magnetic beads overnight at 4 ℃ with gentle rotation. The beads were rinsed twice with lysis buffer and ultrapure water once, boiled in 1 × Lane Marker Sample Buffer, and subjected to SDS-PAGE and western blotting.

### Chromatin immunoprecipitation (Ch-IP) assay

Ch-IP assay was performed using the Simple Ch-IP Kit (Cell Signaling Technology). Approximately 5 × 10^6^ SCLC cells with conditioned treatment for each immunoprecipitation were fixed with formaldehyde. The crosslinked DNA complexes were sheared to lengths of approximately 150–900 base pair fragments and immunoprecipitated with anti-SMAD3, anti-H3K27ac, anti-histone H3 (positive control), or IgG (negative) control antibody overnight at 4 ℃ with rotation. The immunoprecipitated DNA was purified and amplified by PCR. Primers sequences are listed in Supplementary Table 3.

### Luciferase assay

SCLC cells transfected with indicated pcDNA plasmids or pGL3.1 luciferase plasmids by using Lipofectamine 3000 reagent (Invitrogen). After 48 h transfection, cells were subjected to dual luciferase analysis. The results are expressed as a fold induction relative to the cells transfected with the control vector after normalization to Renilla activity.

### Statistics analysis

R software (version 4.2.2) and GraphPad Prism 9(RRID:SCR_002798) were utilized for conducting statistical analyses. Statistical significance was assigned to results with p-values less than 0.05 (ns, *P* ≥ 0.05; *, *P* < 0.05; **,* P* < 0.01; ***, *P* < 0.001). For continuous variables with a normal distribution, the association was assessed using the independent samples t-test. When the continuous data were skewed, the Mann–Whitney U test was applied instead. For categorical variables, the chi-square (X^2^) test was used to analyze relationships when the expected frequencies in each cell of the contingency table were sufficiently large (typically, no more than 20% of cells with expected frequencies < 5 and all expected frequencies ≥ 1). In cases where the sample size was small or expected frequencies were low, violating the assumptions of the chi-square test, Fisher’s exact test was employed as a more appropriate alternative.

## Results

### Elevated HDAC6 expression correlates with SCLC subtypes and poorer survival outcomes

**Fig. 1:**
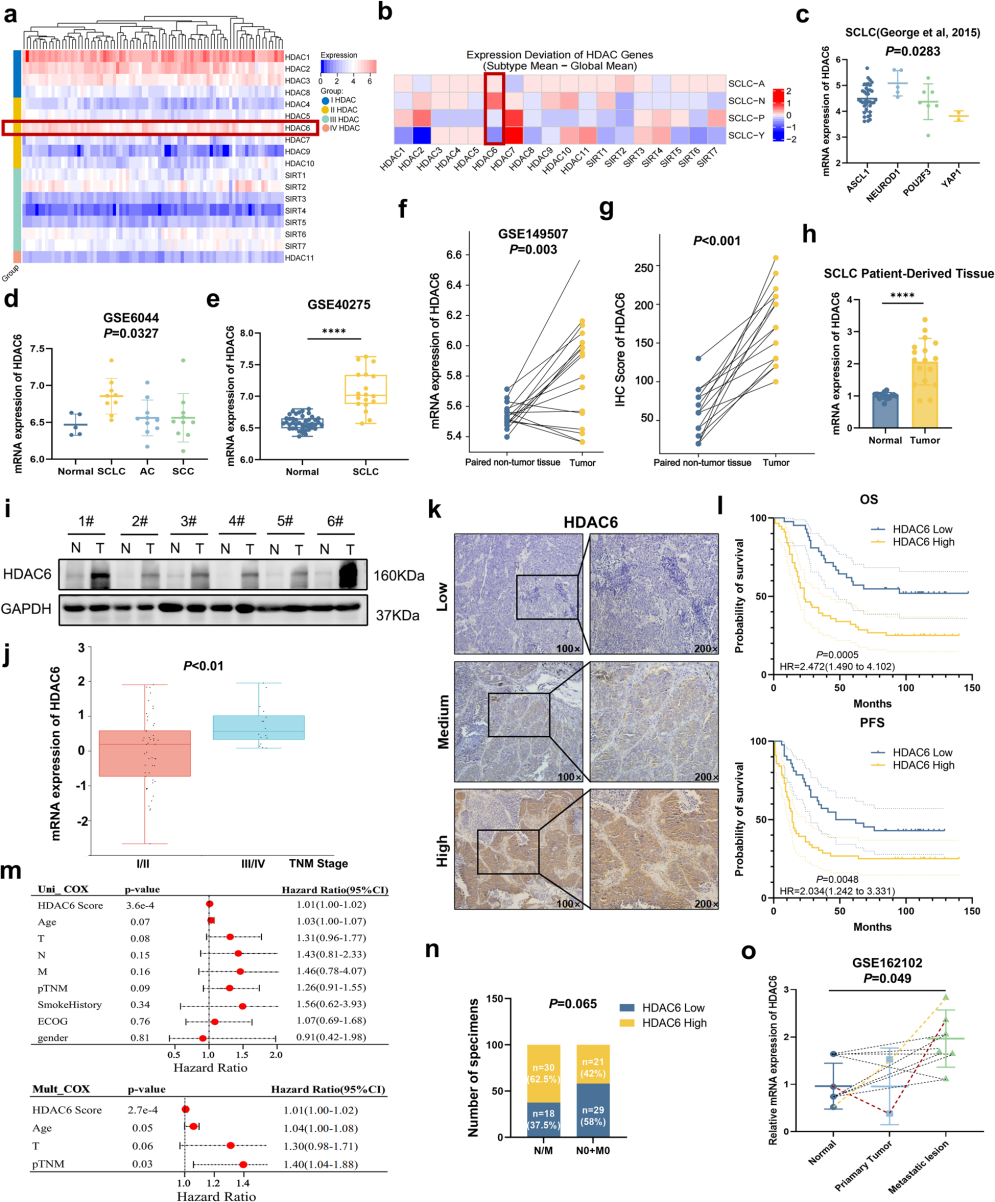
Elevated HDAC6 expression correlates with SCLC subtypes and poorer survival outcomes.** a** mRNA expression profiles of class I-IV HDACs in SCLC based on resected SCLCs (*n* = 81; George et al.). **b** Spearman correlation analysis between the mRNA expression of HDAC families and four key transcription factors of SCLC (n = 81; George et al.). **c** Dot plot showing HDAC6 expression among each subtype in SCLC (n = 81; George et al.). **d** The mRNA expression of HDAC6 across tissue types in GSE6044 dataset. **e** The mRNA expression of HDAC6 between normal and SCLC tissue in GSE40275 dataset. **f** The mRNA expression of HDAC6 between SCLC and paired non-tumor tissue in GSE149507 datasets. **g** IHC score of HDAC6 expression in 15 paired SCLC tumor and adjacent normal tissues from our institutional cohort. **h** HDAC6 mRNA expression levels in patient-derived SCLC and normal tissues based on mRNA sequencing data from our institutional cohort. **i** HDAC6 protein levels in SCLC tumor tissues versus normal tissues. **j** Correlation between HDAC6 mRNA expression and tumor stage in SCLC (George et al.). **k** IHC stain (high/medium/low expression) of HDAC6 in 98 surgically resected SCLC patient samples from our institutional cohort. **l** The KM curves about the correlation between HDAC6 expression and OS and PFS. **m** Univariate and multivariate COX analyses for OS. **n** Association between HDAC6 expression levels (high/low) and metastatic status in SCLC patients in our cohort. **o** The mRNA expression of HDAC6 across normal, primary tumor and metastatic tissue types in GSE162102 dataset. *P < 0.05; **P < 0.01; ***P < 0.001; ****P < 0.0001

To explore the potential involvement of HDAC family members in SCLC progression, we performed expression profiling analysis of existing mRNA sequencing data from 81 surgically resected tumor specimens, primarily from cases of localized SCLC [23]. Among all class II HDAC isoforms, HDAC6 emerged as the only member significantly overexpressed in SCLC tissues (Fig. 1a). Spearman correlation analysis between the mRNA expression of 18 HDAC family members and four key transcription factors of SCLC in George Cohorts revealed a striking subtype bias: HDAC6 expression was markedly enriched in ASCL1- and NEUROD1-positive neuroendocrine subtypes compared to POU2F3- and YAP1-positive subtypes (Fig. 1b). Consistently, dot plot analysis confirmed significant differences in HDAC6 expression across SCLC molecular subtypes (*P* = 0.0283; Fig. 1c). Cross-cohort comparisons further supported these findings. In GSE6044 dataset, HDAC6 mRNA levels were significantly elevated in SCLC tumor tissues compared with normal lung tissues, adenocarcinoma (AC) and squamous cell carcinoma (SCC) (Fig. 1d, *P* = 0.0327). Similar results were obtained from the GSE40275 dataset, where HDAC6 expression was markedly higher in patients with SCLC compared to normal lung tissue (Fig. 1e, *P* < 0.0001). Furthermore, both our analysis of the GSE149507 mRNA sequencing dataset (Fig. 1f, *P* = 0.003) and immunohistochemical (IHC) (Fig. 1g, *P* < 0.001) staining in our institutional SCLC patient cohort consistently demonstrated significantly elevated HDAC6 expression in tumor tissues compared to paired non-tumor samples. Consistent with these findings, both mRNA sequencing and WB analyses of our patient-derived SCLC specimens yielded concordant results (Fig. 1h-i).

Regarding the stage and prognostic value of HDAC6 in SCLC patients, we found that elevated HDAC6 expression correlated with advanced tumor stage in SCLC (George et al., Fig. 1j, *P* < 0.01). We further collected 98 surgically resected SCLC specimens from our institutional cohort for IHC staining and clinical prognosis analysis. The clinical and pathological information is shown in Supplementary Table 1. IHC analysis (Fig. 1k) revealed that patients with high HDAC6 expression had significantly worse overall survival (OS, *P* = 0.0005) and progression-free survival (PFS,* P* = 0.0048) compared to the HDAC6-low group (Fig. 1l). Multivariate Cox regression analysis confirmed HDAC6 as an independent prognostic factor for OS in SCLC (Fig. 1m), with high HDAC6 expression being associated with greater metastatic propensity (Fig. 1n). Analysis of the GSE162102 dataset revealed significantly elevated HDAC6 mRNA expression in metastatic lesions compared to both normal tissues and primary tumors (Fig. 1o, *P* = 0.049). This observation was further corroborated by independent datasets (GSE116977 and GSE161968), which demonstrated higher HDAC6 mRNA levels in either metastatic lesions or SCLC with brain metastases (BM) (Supplementary Fig. 1a-b). Taken together, these results establish HDAC6 as a subtype-biased and metastasis-associated oncogenic driver in SCLC, with elevated expression serving as an independent predictor of poor clinical outcome.

### HDAC6 promotes the proliferation and aggressiveness of SCLC cells

**Fig. 2:**
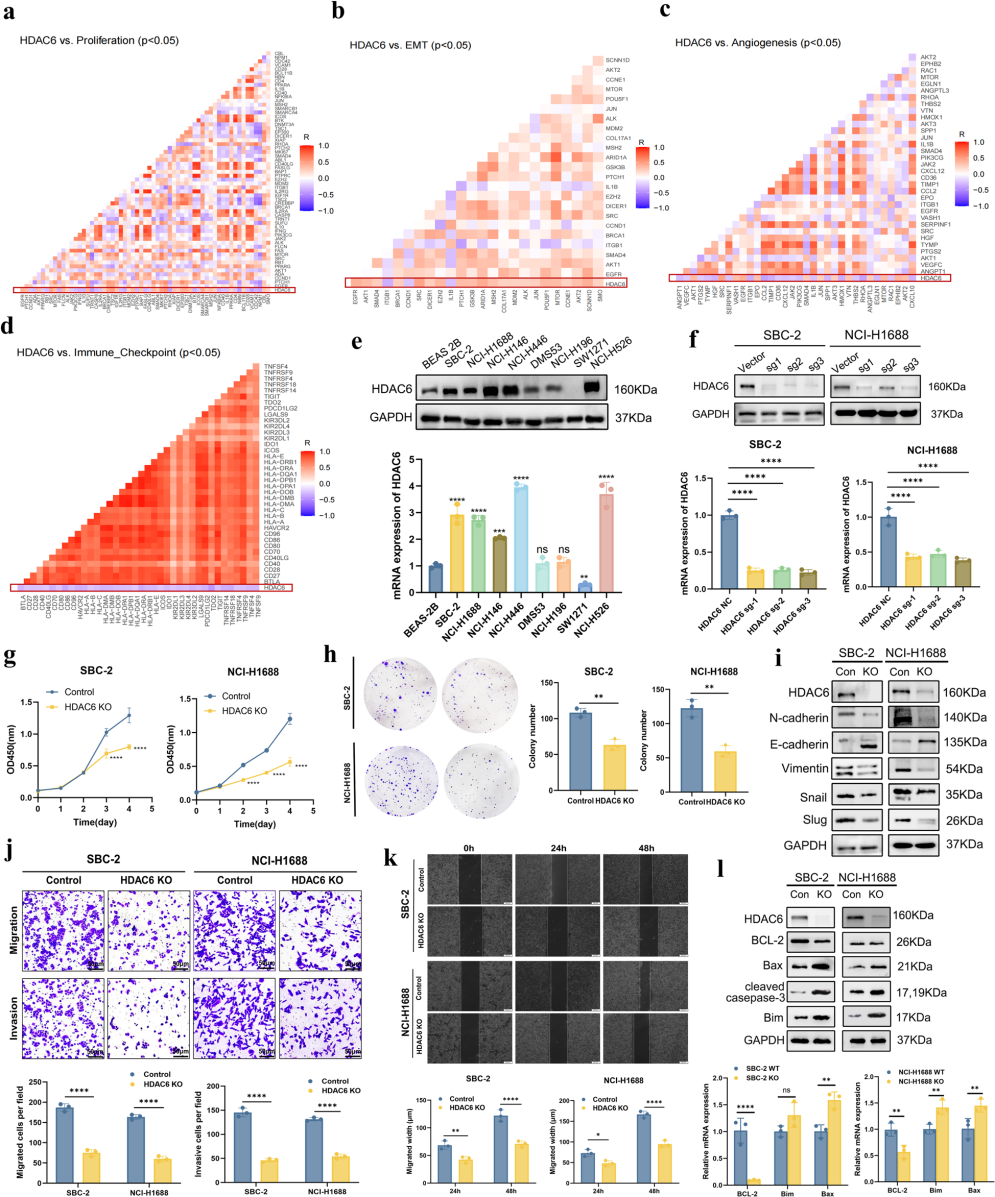
HDAC6 promotes the proliferation and aggressiveness of SCLC cells. **a-d** Co-expression analyses between HDAC6 and gene sets associated with critical oncogenic processes including proliferation (**a**), EMT (**b**), angiogenesis (**c**), and immune checkpoint regulation (**d**) in SCLC (George et al.). **e** The protein and mRNA levels of HDAC6 in normal and SCLC cells. **f** WB and qRT-PCR validated HDAC6 KO efficiency at protein and mRNA levels, respectively. **g-h** CCK8 assay (**g**) and clone formation assay (**h**) between SBC-2 and NCI-H1688 WT cell lines and HDAC6 KO cell lines. **i** WB experiments of EMT-related key molecules in WT cell lines and HDAC6 KO cell lines. **j-k** Transwell assay (**j**) and wound healing assay (**k**) in WT cell lines and HDAC6 KO cell lines. **l** WB and qRT-PCR experiments of apoptotic pathway-related key molecules in WT cell lines and HDAC6 KO cell lines. *P < 0.05; **P < 0.01; ***P < 0.001; ****P < 0.0001

To further investigate the functional role of HDAC6 in SCLC pathogenesis, we first performed comprehensive co-expression analyses between HDAC6 and gene sets associated with critical oncogenic processes, including proliferation, EMT, angiogenesis, and immune checkpoint regulation, using mRNA sequencing data from 81 SCLC tumors (George *et al*.). Our analysis revealed significant positive correlations between HDAC6 expression and genes associated with proliferation and EMT phenotypes (particularly strong with EMT markers), while demonstrating inverse correlations with immune checkpoint molecules (Fig. 2a-d). These findings suggested that HDAC6 might may drive malignant phenotypes and contribute to an immunosuppressive tumor microenvironment. Furthermore, we conducted experiments using WB and qRT-PCR in normal and SCLC cells. Our results showed that HDAC6 was significantly upregulated in most SCLC cells at the protein and mRNA levels, with the most prominent overexpression observed in ASCL1 subtype including NCI-H1688 and NCI-H146 cells, as well as in NEUROD1 subtype such as NCI-H446 cells (Fig. 2e). Based on the baseline HDAC6 expression levels across SCLC cell lines, we selected SBC-2 and NCI-H1688 cells with intermediate HDAC6 expression for subsequent experiments.

We generated two stable HDAC6 KO cell lines using SBC-2 and NCI-H1688 by CRISPR/Cas9 nuclease system (Fig. 2f). Our results revealed that HDAC6 KO significantly reduced the proliferation and clonogenic capacity (Fig. 2g-h). Moreover, we conducted WB experiments to analyze the expression levels of key molecules related to EMT, such as E-cadherin, N-cadherin, vimentin, snail, and slug, as well as key molecules involved in the apoptotic pathway, including the anti-apoptotic gene BCL-2 and the pro-apoptotic genes Bax, Bim, and cleaved-caspase3, in WT and HDAC6 KO cell lines (Fig. 2i and 2 l). Our results revealed that the EMT phenomenon was significantly suppressed, while the apoptotic pathway was activated in both SBC-2 and NCI-H1688 cell lines after the knockout of the HDAC6 gene. These findings were further corroborated by qRT-PCR experiments. Furthermore, we performed transwell and wound healing assays on the two groups of cells. Our findings indicated that the migration and invasion abilities of SCLC cells were significantly decreased after the knockout of HDAC6 (Fig. 2j-k). To comprehensively characterize the functional role of HDAC6 in SCLC, we performed HDAC6 OE in both SBC-2 and NCI-H1688 cells, as demonstrated in Supplementary Fig. 1c. Subsequent functional assays in both WT and HDAC6 OE cell lines including CCK8, colony, Transwell, wound healing, and EMT marker analysis by WB consistently demonstrated that HDAC6 OE enhanced malignant phenotypes in SCLC (Supplementary Fig. 1d-h). To further validate the pivotal role of HDAC6 in SCLC malignancy, we performed genetic rescue experiments by re-introducing HDAC6 via OE lentiviral in HDAC6 KO cell lines (Supplementary Fig. 1i). Subsequent cell function experiments demonstrated partial to complete reversal of the attenuated malignant phenotypes observed in KO models, providing direct evidence that HDAC6 was functionally required for SCLC aggressiveness (Supplementary Fig. 1j-m and 2a). Together, these results establish HDAC6 as a pivotal driver of proliferation, EMT, and invasive potential in SCLC cells.

In addition, we initially determined the 24 h and 48 h IC50 values of Tubastatin A in SBC-2 and NCI-H1688 cell lines (Supplementary Fig. 2b). Based on these results, we ultimately selected 25 μM for SBC-2 and 20 μM for NCI-H1688 for subsequent functional assays. We performed CCK-8 proliferation assays and colony formation assays in both control and drug-treated cell lines. Our results revealed that the proliferation and clone formation abilities of the drug-treated cell lines were significantly reduced (Supplementary Fig. 2c-d). Moreover, we conducted WB experiments to analyze the expression levels of key molecules related to EMT and the apoptotic pathway. Our results revealed that Tubastatin A treatment suppressed EMT while activating the apoptotic pathway in both SBC-2 and NCI-H1688 cells (Supplementary Fig. 2e-g). These findings were further corroborated by qRT-PCR experiments. Furthermore, we assessed the migratory and invasive potential of SCLC cells using transwell and wound healing assays, which revealed markedly reduced migration and invasion upon Tubastatin A treatment (Supplementary Fig. 2 h-j). Taken together, these results demonstrate that pharmacological inhibition of HDAC6 by Tubastatin A suppresses proliferative, invasive, and EMT-driven malignant phenotypes in SCLC cells, thereby recapitulating the effects observed with genetic ablation of HDAC6.

### HDAC6 promotes SCLC progression and metastatic potential

**Fig. 3:**
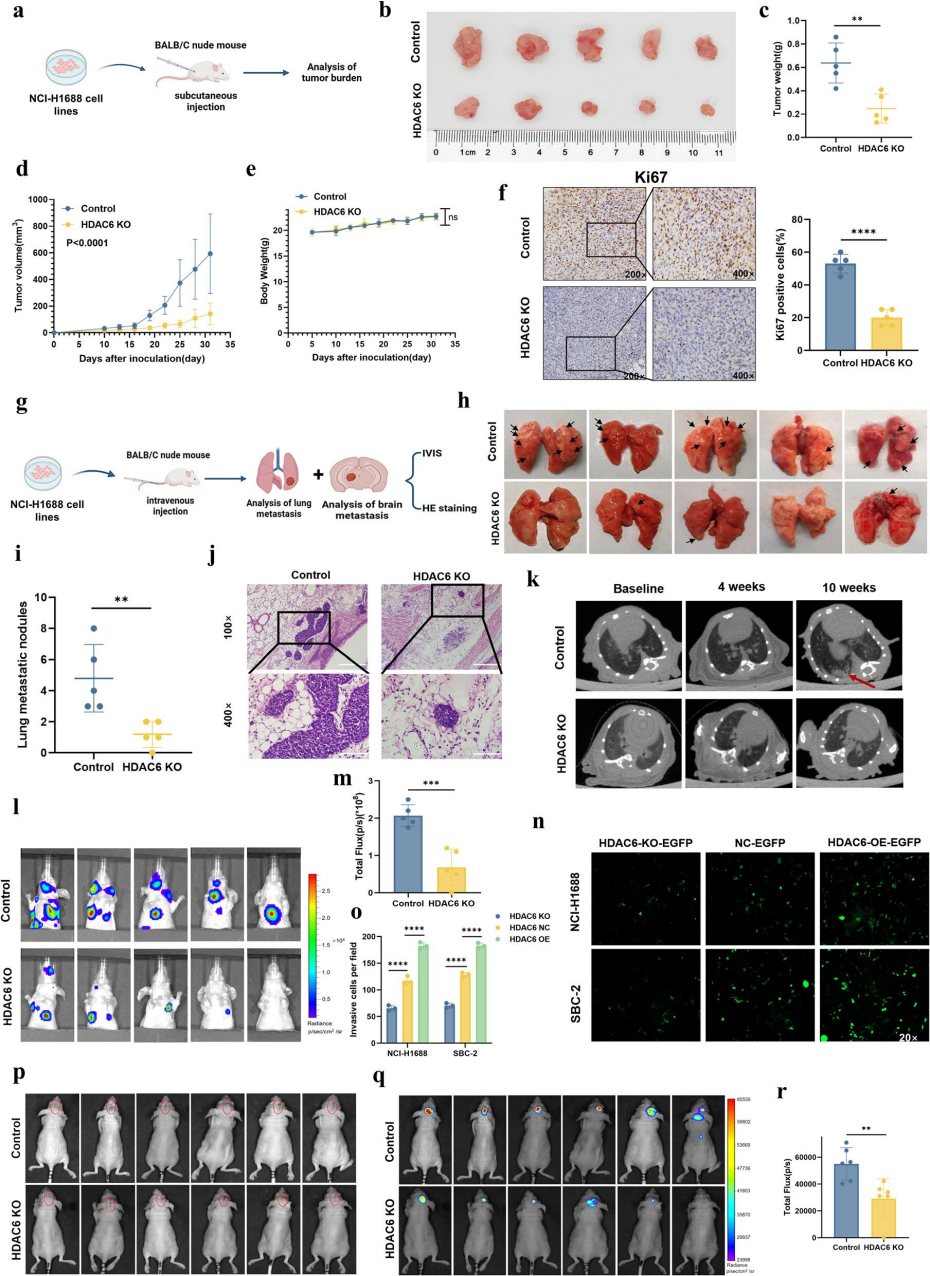
HDAC6 promotes SCLC progression and metastatic potential.** a-b** BALB/C nude mice were implanted with HDAC6 KO or control NCI-H1688 cells. **c-d** Comparison of tumor weight (**c**) and volume (**d**) between two groups. **e** The body weight measurements of mice were taken every 3 days. **f** Percentage of Ki67-positive cells between two groups. **g** Metastatic burden comparison by tail vein injection of luciferase-labeled NCI-H1688 cells (NC vs HDAC6 KO). **h-i** Comparison of pulmonary metastatic nodule counts between groups. **j** Comparison of pulmonary metastatic lesion areas by HE staining between groups. **k-m** Longitudinal monitoring via pulmonary CT (**k**) and IVIS imaging (**l-m**). **n–o** Penetration capacity of SCLC cells across BBB model under different HDAC6 manipulations. **p** Bright-field images of intracranial orthotopic injection in the two groups. **q-r** Longitudinal monitoring via IVIS imaging (**q-r**). *P < 0.05; **P < 0.01; ***P < 0.001; ****P < 0.0001

Our in vitro functional studies identified HDAC6 as a critical regulator of SCLC proliferation and invasion. To validate these findings in vivo, we subcutaneously injected NCI-H1688 WT and HDAC6 KO cells into BALB/c nude mice (Fig. 3a). After one month, xenograft tumors were excised and analyzed, revealing that HDAC6 KO tumors exhibited significantly reduced tumor weight (*P* < 0.01) and tumor volume (*P* < 0.0001) compared to controls (Fig. 3b-d), while no significant difference in body weight was observed between groups (Fig. 3e). Further IHC analysis of paraffin-embedded tumor sections demonstrated a marked decrease in Ki67-positive cells in HDAC6 KO tumors (Fig. 3f), collectively indicating that HDAC6 depletion substantially inhibited SCLC cell proliferation in vivo.

To further investigate the role of HDAC6 in SCLC invasion and metastasis in vivo, we intravenously injected luciferase-labeled NCI-H1688 cells (NC control and HDAC6 KO groups) into the tail veins of BALB/c nude mice and monitored metastatic progression (Fig. 3g). Ten weeks later, mice in the KO group displayed significantly fewer metastatic nodules on lung surfaces (Fig. 3h-i) and reduced lesion areas by HE staining (Fig. 3j). Longitudinal monitoring with pulmonary CT and IVIS imaging revealed decrease in metastatic burden in KO mice (Fig. 3k-m), with control mice exhibiting more severe lung and brain metastases (BM). To mechanistically investigate the contribution of HDAC6 to BM, we established an optimized Blood–Brain Barrier (BBB) model using HBMEC/NHA co-culture (Supplementary Fig. 2 k), which showed peak barrier function at day 5, as evidenced by positive 4-h leakage testing (Supplementary Fig. 2 l), minimal sodium fluorescein permeability (40.3%) (Supplementary Fig. 2 m) and maximal tight junction proteins expression (Claudin-5/Occludin/ZO-1) (Supplementary Fig. 2n-o). Using this optimized 5-day BBB model, we seeded EGFP-labeled SCLC cells (HDAC6 KO, NC, and OE) in the upper chamber and quantified transmigrated cells in the lower chamber after 2 h via confocal microscopy. As shown in Fig. 3n-o, HDAC6 KO significantly reduced SCLC cell penetration through the BBB model, whereas HDAC6 OE enhanced transmigration. These in vitro findings demonstrated that HDAC6 functionally enhanced SCLC cell penetration across the BBB, suggesting that it may play an important role in promoting BM. To further investigate the role of HDAC6 in SCLC metastasis, we established brain and systemic metastasis models via intracranial or intracardiac injection of tumor cells. As shown in Fig. 3p, luciferase-expressing NCI-H1688 HDAC6 WT and KO cells were stereotactically injected into the mouse striatum. Subsequent longitudinal IVIS imaging revealed a significantly reduced brain metastatic burden in the KO group (Fig. 3q-r). Similarly, intracardiac injection of these cells followed by IVIS monitoring showed attenuated systemic metastasis to organs like the lungs and brain in HDAC6 KO mice (Supplementary Fig. 3a-b). These findings indicate that HDAC6 plays a critical role in SCLC metastasis and malignant progression.

### Spatial correlation between HDAC6 expression and metastasis-related markers in SCLC patients

**Fig. 4:**
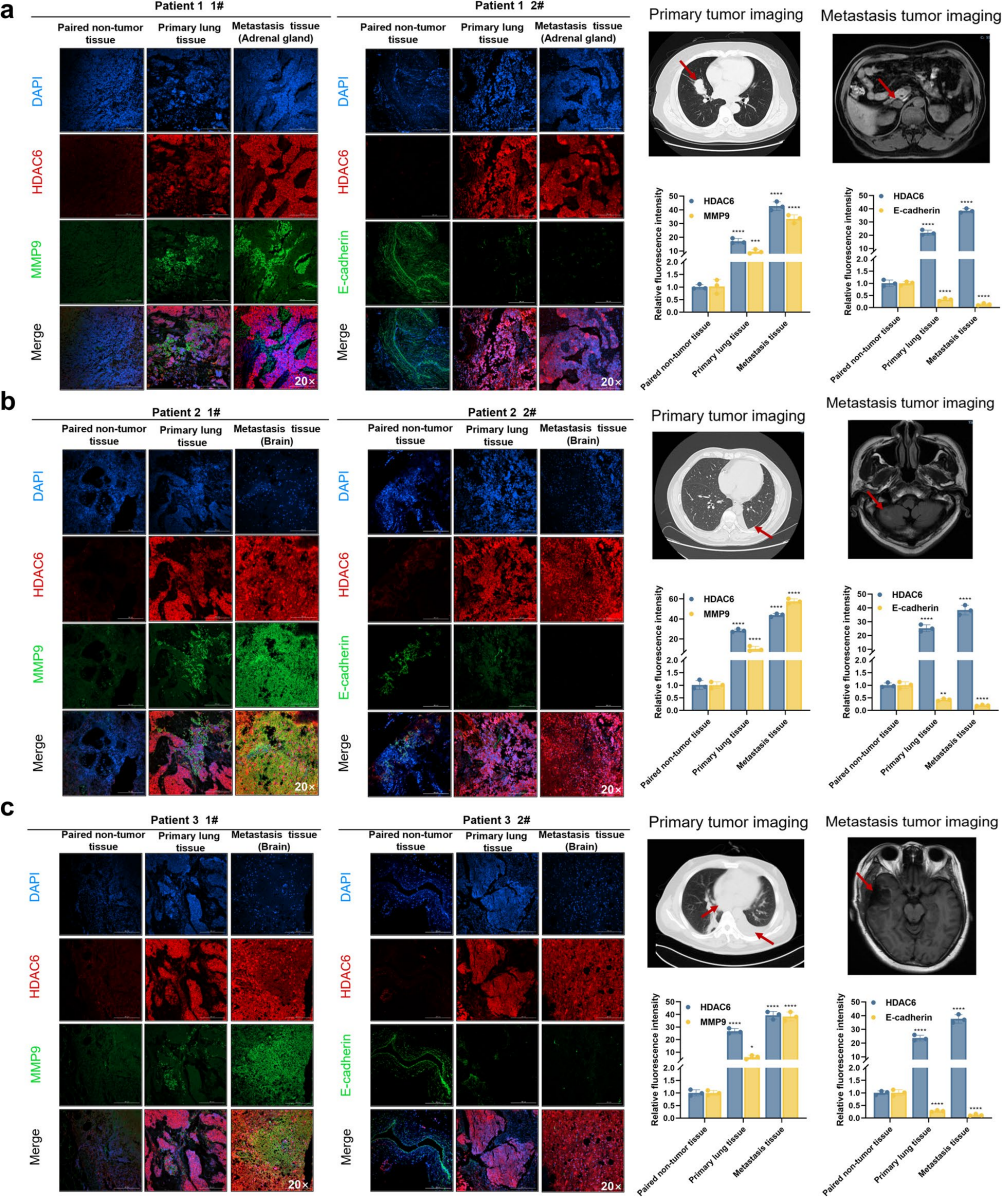
Spatial correlation between HDAC6 expression and metastasis-related markers in SCLC patients. **a**-**c** IF staining on matched paired non-tumor, primary lung tumor, and metastatic tissues from three SCLC patients, typical imaging images of primary and metastatic lesions and comparison of fluorescence intensity among all three tissue types. *P < 0.05; **P < 0.01; ***P < 0.001; ****P < 0.0001

To further clarify the correlation between HDAC6 expression and metastatic potential in SCLC, we conducted multiplex immunofluorescence (IF) staining on matched paired non-tumor, primary lung tumor, and metastatic tissues from three SCLC patients (Fig. 4a-c). Quantitative analysis revealed that HDAC6 expression was positively correlated with MMP9, which showed the highest expression in metastatic lesions, but inversely correlated with E-cadherin, which displayed the lowest expression in metastatic tissues. Differences in fluorescence intensity across the three tissue types were statistically significant (all *P* < 0.05). Typical imaging images of primary and metastatic lesions are also shown in Fig. 4a-c. Collectively, these findings indicate that HDAC6 expression is spatially associated with metastatic biomarkers and may contribute to the acquisition of invasive phenotypes in SCLC.

### HDAC6-mediated S100A2 deacetylation activates TGF-β signaling to promote EMT in SCLC

**Fig. 5:**
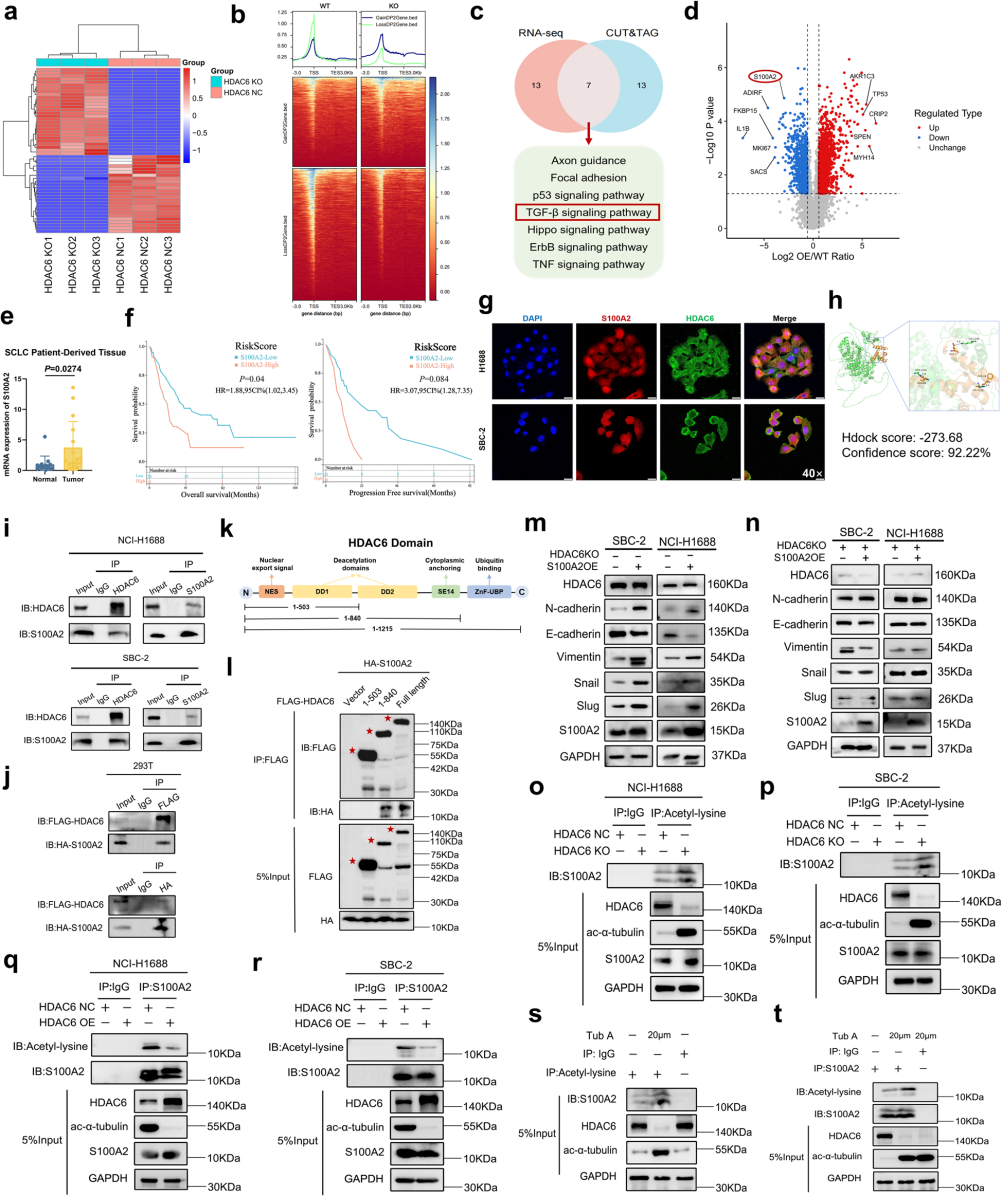
HDAC6-mediated S100A2 deacetylation activates TGF-β signaling to promote EMT in SCLC.** a** Heatmap of differentially expressed genes between NCI-H1688 HDAC6 WT and KO groups from mRNA-seq. **b** Heatmap of genome wide H3K27ac CUT&TAG analysis revealed enrichment of H3K27ac peaks around TSS (± 1 kb) of genes that were differentially expressed d in two groups. **c** Venn diagram showing overlap of KEGG passways between two data. **d** Volcano plot of differentially acetylated proteins in NCI-H1688 HDAC6 WT vs OE from acetyl-proteomics. **e** S100A2 mRNA expression levels in patient-derived SCLC and normal tissues based on mRNA sequencing data from our institutional cohort. **f** The KM curves about the correlation between HDAC6 expression and OS and PFS (George et al.). **g** Immunofluorescence co-localization of HDAC6/S100A2 in SBC-2 and NCI-H1688 cells. **h** Molecular docking results of HDAC6-S100A2 interaction. **i** Co-IP/WB analysis of HDAC6-S100A2 interaction in SBC-2 and NCI-H1688 cells using reciprocal bait antibodies. **j** Reciprocal Co-IP/WB with FLAG/HA-tagged proteins in HEK293T cells validates HDAC6-S100A2 interaction. **k** Schematic diagram (not drawn to scale) of HDAC6 and three HDAC6 deletion mutants. **l** HEK293T cells were transfected with plasmids encoding HA-tagged S100A2 and various FLAG-tagged HDAC6 truncated mutants. Anti-FLAG immunoprecipitation was western blotted with antibodies specific for Flag. **m** WB experiments of EMT markers in WT and S100A2 OE cell lines. **n** WB analysis of EMT markers in S100A2 WT and OE cells with HDAC6 KO background. **o-p** Co-IP with Acetyl-lysine antibody comparing S100A2 levels between HDAC6 NC and KO groups in NCI-H1688 (**o**) and SBC-2 (**p**) cells. **q-r** Co-IP with S100A2 antibody comparing Acetyl-lysine levels between HDAC6 NC and OE groups in NCI-H1688 (**q**) and SBC-2 (**r**)cells. **s-t** Co-IP with Acetyl-lysine (**s**) and S100A2 (**t**) antibodies comparing acetylation level of lysine residues on S100A2 between control and Tub A-treated groups in NCI-H1688 cells

To investigate the mechanism by which HDAC6 regulates malignant phenotypes in SCLC, we performed RNA-seq using NCI-H1688 HDAC6 WT and KO cell lines. With thresholds set at |log2FC|≥ 2 and Q-value < 0.0001, we identified 974 significantly downregulated genes and 785 significantly upregulated genes upon HDAC6 KO (Supplementary Fig. 3c). Heatmap analysis of differentially expressed genes revealed pronounced downregulation of proliferation- and metastasis-associated genes (Fig. 5a). We further analyzed the genome wide changes in H3K27ac of HDAC6 WT and KO cell lines of NCI-H1688. Hence, we carried out CUT&TAG analysis in these cell lines and found H3K27ac peaks around TSS (± 1 kb) of genes that were differentially expressed in two groups (Fig. 5b). Additionally, as shown in Supplementary Fig. 3 d, the functional distribution differences of both upregulated and downregulated differentially peaked regions (DPs) were predominantly localized within 1 kb of promoter regions. We further analyzed the expression differences of EMT-related markers through RNA-seq data. As shown in Supplementary Fig. 3e, the epithelial marker E-cadherin was significantly upregulated following HDAC6 KO, while mesenchymal markers (Vimentin, Slug, N-cadherin) showed marked downregulation, indicating attenuated EMT progression. We specifically analyzed differentially expressed transcription factors and identified that SNAI2 (Slug), a master regulator of EMT, was significantly downregulated following HDAC6 KO (Supplementary Fig. 3f). Integrated analysis of RNA-seq and CUT&TAG data revealed that HDAC6-associated differentially expressed genes were significantly enriched in pathways related to cell proliferation and migration (GO analysis) (Supplementary Fig. 3 g-h). RNA-seq results revealed that 6 out of the top 20 enriched pathways were associated with EMT. Intersection of the top 20 KEGG pathways from both sequencing datasets identified seven overlapping pathways, including two EMT-associated pathways (Fig. 5c). Based on the combined metrics of Risk Ratio (RR) and* P*-value, we prioritized the TGF-β signaling pathway for further investigation. These findings suggested that HDAC6 likely promoted SCLC malignancy through TGF-β pathway-mediated EMT regulation. These results suggested that HDAC6 promotes EMT in SCLC, at least in part, through TGF-β pathway activation.

To explore how HDAC6 activates the TGF-β pathway, we performed quantitative acetyl-proteomics sequencing in NCI-H1688 HDAC6 WT and OE cell lines. The heatmap of differentially acetylated proteins between groups was shown in Supplementary Fig. 4a, revealing that HDAC6-mediated deacetylation primarily targets lysine residues (Supplementary Fig. 4b). Functional enrichment analysis demonstrated that HDAC6-regulated proteins were predominantly involved in biological regulation and cellular metabolism processes (GO analysis) (Supplementary Fig. 4c), with KEGG pathway enrichment highlighting lysine degradation, cytokine—cytokine receptor interaction, and p53 signaling pathways (Supplementary Fig. 4 d). The HDAC6-related proteins with significant intergroup changes were visualized using volcano plots (Fig. 5d). Among the candidate substrates, we observed that acetylated S100A2 was significantly downregulated in the HDAC6 OE group. Based on the |log2FC| and P-values, we identified S100A2 as a novel deacetylation substrate of HDAC6. S100A2 was upregulated in various types of tumors and serves as a risk factor for OS and PFS in multiple cancers (Supplementary Fig. 4e-f). Based on prior literature [26, 27], S100A2 is known to activate TGF-β/SMAD signaling. Collectively, these findings establish S100A2 as a novel substrate of HDAC6, whose deacetylation promotes TGF-β/SMAD-driven EMT and thereby enhances the invasive capacity of SCLC cells.

Based on RNA sequencing data from our cohort revealed SCLC tumors exhibited higher expression levels of S100A2 expression compared to adjacent normal tissue (Fig. 5e). As shown in Fig. 5f, elevated S100A2 was significantly associated with worse OS in SCLC patients (*P* = 0.04) and showed a trend towards worse PFS (*P* = 0.084). To validate the direct interaction between HDAC6 and S100A2, we first performed immunofluorescence staining, revealing their co-localization in SCLC cell lines (Fig. 5g). Furthermore, molecular docking analysis of HDAC6 and S100A2 protein structures yielded an Hdock score of −273.68 and a confidence score of 92.22%, strongly supporting their high-probability interaction (Fig. 5h). Subsequently, reciprocal co-immunoprecipitation (Co-IP) assays in NCI-H1688 and SBC-2 cells, using HDAC6 and S100A2 as bait antibodies respectively, confirmed endogenous binding between HDAC6 and S100A2 (Fig. 5i). Consistently, for exogenous validation, we co-transfected FLAG-tagged HDAC6 and HA-tagged S100A2 plasmids into HEK293T cells, and reciprocal Co-IP with anti-FLAG/HA antibodies demonstrated their in vitro binding (Fig. 5j). To further clarify which specific segment of HDAC6 binds to S100A2, we conducted HDAC6 truncation mutation experiments. Figure 5k showed a schematic diagram (not drawn to scale) of HDAC6 and three HDAC6 deletion mutants. NES: nuclear export signal. DD1 and DD2: deacetylase domain 1 and 2. SE14: cytoplasmic anchoring. ZnF-UBP: ubiquitin-binding zinc finger. HEK293T cells were transfected with plasmids encoding HA-tagged S100A2 and either FLAG- tagged full length (1–1215) or deletion mutants of HDAC6. Full-length HDAC6 and a C-terminal HDAC6 deletion mutant (1–840) bound S100A2 (Fig. 5l). Conversely, the HDAC6 mutant (1–503) lacking both the C-terminus and the second deacetylase domain (DD2) completely lost its ability to bind S100A2, suggesting that DD2 of HDAC6 was essential for S100A2 binding.

To further clarify whether HDAC6 functioned as a key upstream molecule of S100A2 in SCLC, we transfected the S100A2 OE plasmid under the backgrounds of HDAC6 WT and KO, respectively. Supplementary Fig. 4 g-h presented the validation of the transfection efficiency of S100A2 OE. We performed WB analysis of EMT markers in the aforementioned two sets of cell lines. The results revealed that in the two cell lines with HDAC6 WT, the S100A2 OE group significantly enhanced the EMT phenomenon in SCLC (Fig. 5m). However, in the two cell lines with an HDAC6 KO background, S100A2 OE did not significantly promote EMT (Fig. 5n). These findings suggested that HDAC6 was an important upstream gene for S100A2 to exert its function, which indicated that the acetylation activity of S100A2 might influence its role as an oncogene in SCLC. Next, we conducted further experiments to verify that HDAC6 could regulate the acetylation level of S100A2. We performed Co-IP experiments using an acetyl-lysine antibody as the bait antibody in cell lines with HDAC6 NC and KO. The results showed that more S100A2 protein was pulled down in the HDAC6 KO group (Fig. 5o-p). Similarly, we conducted Co-IP experiments using an S100A2 antibody as the bait antibody in cell lines with HDAC6 NC and OE, and observed that less acetyl-lysine protein was pulled down in the OE group (Fig. 5q-r). Additionally, we administered the HDAC6 inhibitor Tub A at a concentration of 20 μM to the NCI-H1688 cell line and performed Co-IP assays using the aforementioned two antibodies (Fig. 5s-t). We found that the acetylation level of lysine residues on S100A2 was higher in the drug-treated group. Collectively, these results demonstrate that HDAC6 directly deacetylate S100A2, and thereby regulating its acetylation status and oncogenic function in SCLC.

### Acetylation status of S100A2 at lysine 27 site modulates the stability and nuclear translocation of downstream p-SMAD2/3 and SMAD2/3/4 complexes

**Fig. 6:**
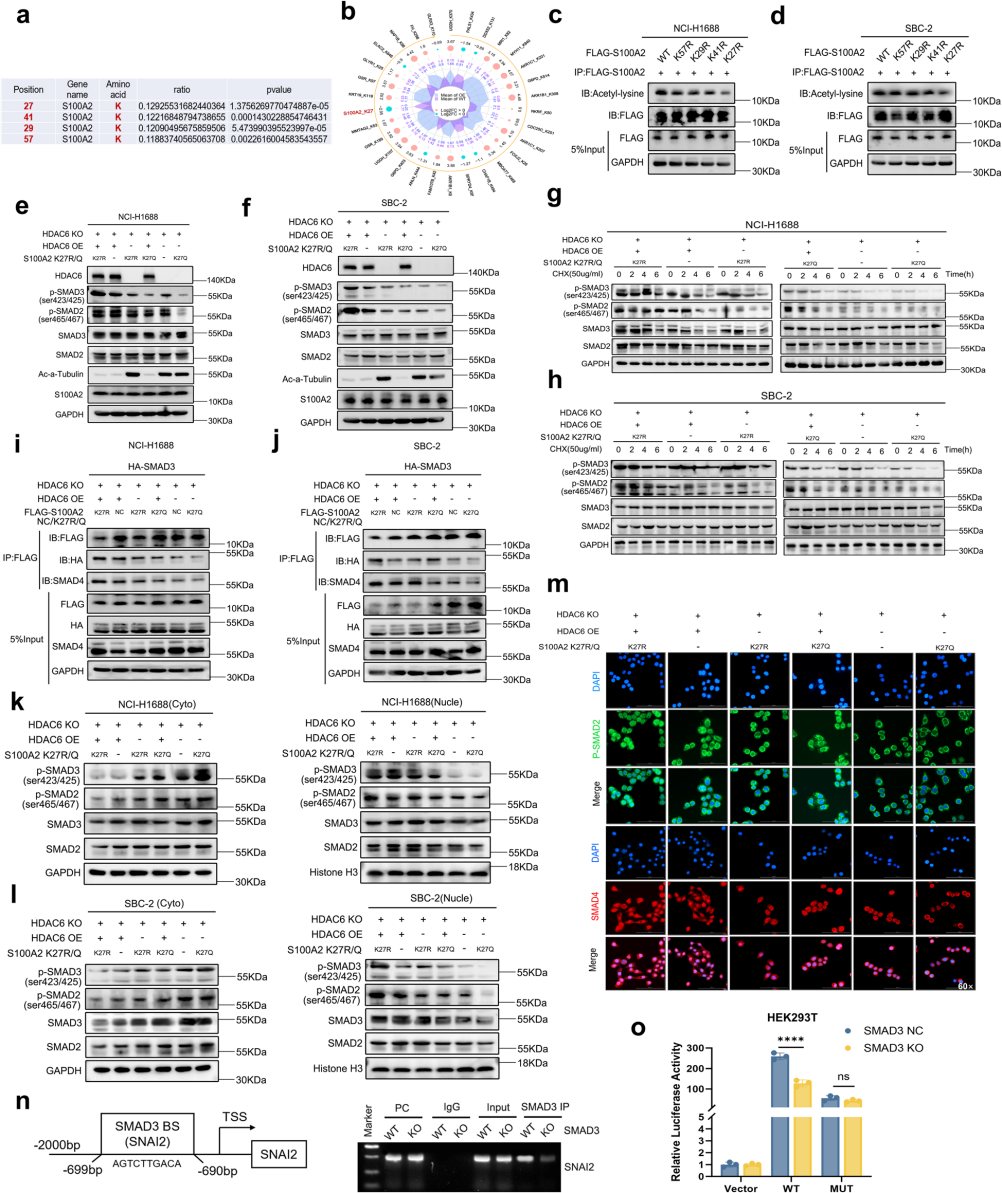
Acetylation status of S100A2 at lysine 27 site modulates the stability and nuclear translocation of downstream p-SMAD2/3 and SMAD2/3/4 complexes.** a** HDAC6-targeted deacetylation sites in S100A2. **b** Top 30 acetylated proteins and sites in NCI-H1688 HDAC6 WT vs OE visualized by radar plot. **c-d** FLAG-tagged S100A2 WT, K57R, K29R, K41R and K27R mutants were expressed in NCI-H1688 and SBC-2 cells by transient transfection. Anti-acetyl-lysine immuno-precipitates were western blotted with antibodies specific to FLAG. **e–f** Expression of p-SMAD2/3 downstream of S100A2 acetylation site mutants under different HDAC6 expression levels. **g-h** Stability of p-SMAD2/3 under different HDAC6 levels and S100A2 acetylation mutants after CHX treatment. **i-j** FLAG-tagged S100A2 NC, K27R, K27Q mutants and HA-tagged SMAD3 were expressed in NCI-H1688 and SBC-2 cells by transient transfection. Anti-HA and SMAD4 immuno-precipitates were western blotted with antibodies specific to FLAG. **k-l** Nuclear accumulation of p-SMAD2/3 under different HDAC6 levels and S100A2 acetylation mutants. **m** IF analysis of nuclear p-SMAD2 and SMAD4 levels under varying HDAC6 expression and S100A2 acetylation mutants in NCI-H1688 cell line. **n** Ch-IP assay was performed to validate changes in binding ability of SMAD3 to promoter of SNAI2 under SMAD3 WT or SMAD3 KO in HEK293T cell line. PC: positive control **o** Dual luciferase assay was performed to determine the promoter activity in HEK293T cell line

According to the quantitative acetyl proteomics sequencing results, HDAC6 deacetylated S100A2 at four specific lysine sites (K27, K41, K29, and K57), as shown in Fig. 6a, with K27 being the most prominent target (Fig. 6b). To validate these findings, we transfected NCI-H1688 and SBC-2 cell lines with FLAG-tagged plasmids encoding WT S100A2 or its K27R, K41R, K29R, and K57R point mutants (where arginine [R] substitution mimics a constitutively deacetylated state). Co-IP was performed using an anti-FLAG antibody to assess the levels of acetylated lysine proteins recruited in each group. The results demonstrated that the K27R mutant exhibited the lowest level of acetylated lysine proteins, indicating that K27 was the primary deacetylation site of HDAC6 on S100A2 (Fig. 6c-d). Based on these findings, subsequent experiments in this study primarily focused on the K27 site. Under HDAC6 KO conditions, with or without HDAC6 OE rescue, we transfected S100A2 K27R or K27Q mutant plasmids (where K27Q mimics a constitutively acetylated state via lysine-to-glutamine substitution) into cells with varying HDAC6 expression levels. As shown in Fig. 6e-f, the experiment comprised six groups. SMAD2/3/4 and p-SMAD2/3 are canonical components of the TGF-β/SMAD signaling pathway. S100A2 binds to the SMAD2/3/4 complex, protecting its phosphorylation sites from phosphatase-mediated degradation while facilitating nuclear translocation. Although S100A2, SMAD2, and SMAD3 expression levels remained comparable across all six experimental groups, we observed the highest expression of p-SMAD3 (Ser423/425) and p-SMAD2 (Ser465/467) in the HDAC6-expressing group with S100A2 K27R mutation (constitutively deacetylated state), indicating maximal downstream activation when S100A2 was most active. Conversely, the HDAC6 KO group with S100A2 K27Q mutation (constitutively acetylated state) showed the lowest p-SMAD2/3 levels (Fig. 6e-f).

To further examine how S100A2 acetylation status affected p-SMAD2/3 stability, we performed cycloheximide (CHX, 50 μg/mL) chase assays, collecting protein samples at 0, 2, 4, and 6 h. While total SMAD2 and SMAD3 degradation was minimal across all groups, p-SMAD3 (Ser423/425) and p-SMAD2 (Ser465/467) were most stable and degraded slowest in the HDAC6-expressing group with S100A2 deacetylation-mimicking K27R. In contrast, these phosphorylated forms degraded most rapidly in the HDAC6 KO group with S100A2 acetylation-mimicking K27Q (Fig. 6g-h). These findings demonstrated that S100A2 acetylation status and activity directly regulated its functional potency on downstream molecules, with a more pronounced effect on p-SMAD3 than p-SMAD2. We therefore hypothesized that S100A2 primarily interacted with SMAD3 within the SMAD2/3/4 complex. To test this, we conducted Co-IP experiments using HA-tagged SMAD3 and FLAG-tagged S100A2 mutants. The results showed significantly greater recruitment of HA-SMAD3 and SMAD4 proteins in the HDAC6-expressing S100A2 K27R group, confirming that S100A2 exhibited strongest binding affinity with SMAD3 when maintained in its deacetylated active state (Fig. 6i-j).

In addition, to further investigate the nuclear translocation of p-SMAD2/3 and SMAD2/3/4 complexes across different experimental groups, we performed nuclear-cytoplasmic fractionation assays. The results demonstrated that the HDAC6-expressing group with S100A2 K27R mutation (constitutively deacetylated state) exhibited the highest nuclear accumulation of p-SMAD3 (Ser423/425) and p-SMAD2 (Ser465/467), while the HDAC6 KO group with S100A2 K27Q mutation (constitutively acetylated state) showed the lowest nuclear levels of these phosphorylated proteins (Fig. 6k-l). Furthermore, IF staining using p-SMAD2 (green fluorescence) and SMAD4 (red fluorescence) antibodies in NCI-H1688 cells revealed a progressive decrease in nuclear localization of these molecules, with the strongest signal in the HDAC6^+^/S100A2 K27R group and the weakest in the HDAC6 KO/S100A2 K27Q group (Fig. 6m). These findings collectively indicated that the deacetylation status and activity of S100A2 significantly influenced nuclear translocation of downstream molecules, particularly affecting the nuclear import of activated p-SMAD2/3, thereby amplifying TGF-β signaling in SCLC.

The SMAD2/3/4 complex translocated into the nucleus to regulate the transcription of EMT-related transcription factors. It is known that SMAD2 lacks a DNA-binding domain and cannot directly bind DNA independently, while SMAD4 itself has no intrinsic transcriptional activity and primarily functions as a co-activator. Previous studies have shown that SMAD3 can directly bind to the promoters of multiple EMT-related transcription factors, including SNAI1 (Snail) and SNAI2 (Slug), although the precise binding sites remain poorly characterized. To investigate this, we knocked out SMAD3 in NCI-H1688 and SBC-2 cell lines (Supplementary Fig. 5a-b) and observed a significant downregulation of SNAI2 and other EMT-related markers upon SMAD3 deletion (Supplementary Fig. 5c), suggesting that SMAD3 was required for EMT transcriptional programs. Furthermore, using the JASPAR database, we identified a putative SMAD3-binding motif within the *SNAI2* promoter region (Fig. 6n, left). Ch-IP DNA electrophoresis assays confirmed that SMAD3 KO significantly reduced the binding affinity of SMAD3 to this predicted site in the *SNAI2* promoter (Fig. 6n, right). To further validate these findings, we performed dual-luciferase reporter assays in HEK293T cells. The results showed that the full-length *SNAI2* promoter (WT) exhibited significantly reduced luciferase activity upon SMAD3 deletion, whereas mutation of the predicted binding site abolished the difference in luciferase activity between the two groups (Fig. 6o), confirming its functional relevance. Together, these results demonstrate that SMAD3 directly regulates *SNAI2* transcription by binding its promoter, thereby driving EMT progression in SCLC.

### The synergistic regulation of tumor growth and metastasis in SCLC by HDAC6 and CSF1R inhibitors

**Fig. 7:**
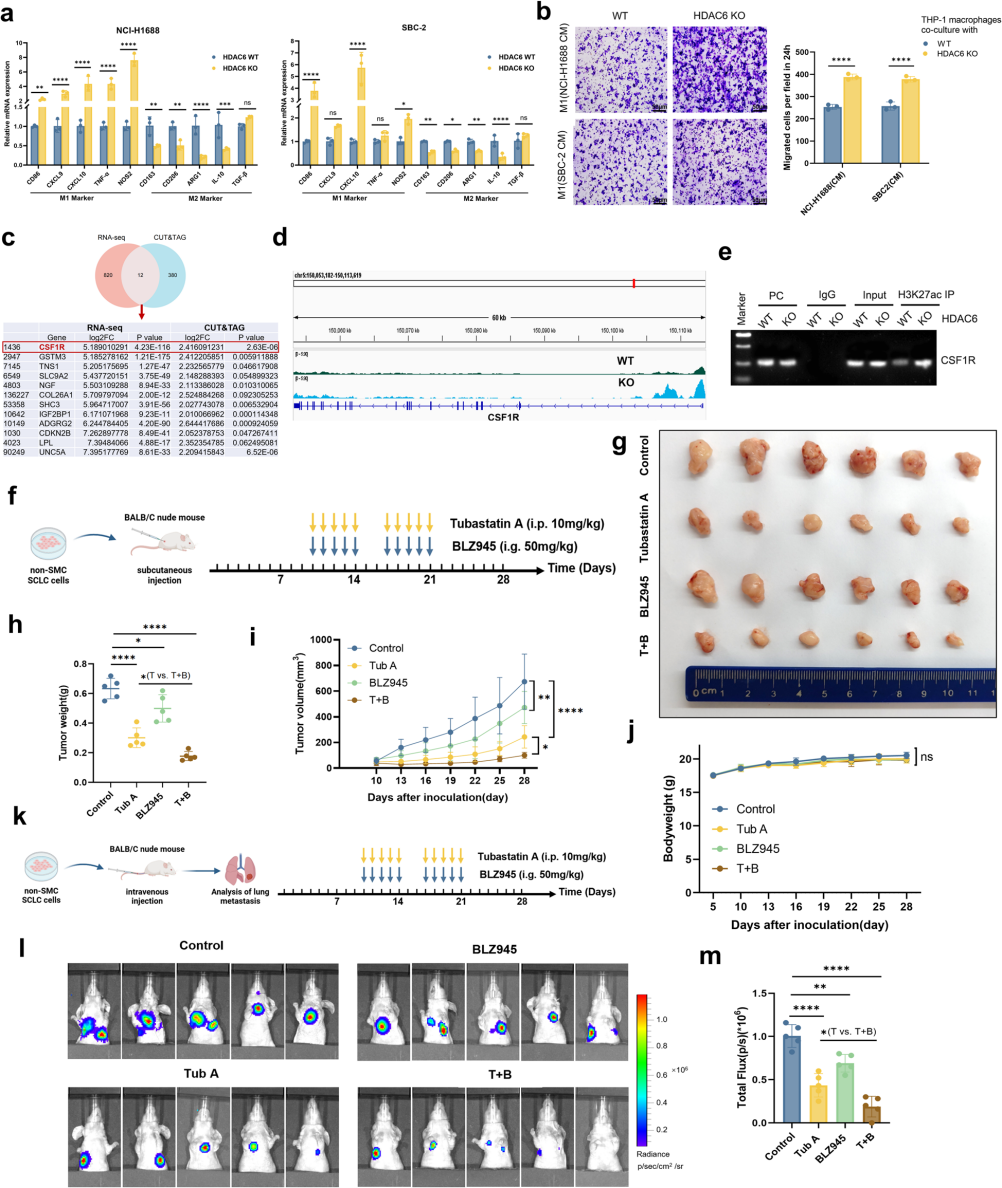
The synergistic regulation of tumor growth and metastasis in SCLC by HDAC6 and CSF1R inhibitors.** a** Effect of the HDAC6 gene on macrophage polarization in NCI-H1688 and SBC-2 cells. **b** Effect of HDAC6 gene on macrophage migration in NCI-H1688 and SBC-2 cells. CM: Conditioned Medium **c** Venn diagram of genes significantly upregulated in HDAC6 KO group compared to HDAC6 WT group from mRNA-seq and CUT&TAG sequencing data. **d** CUT&TAG sequencing analysis performed in HDAC6 KO cells showed increased H3K27ac peaks near the TSS of the CSF1R gene. **e** Ch-IP assay: HDAC6 binding at CSF1R promoter (H3K27ac-marked sites) in NCI-H1688 cells (HDAC6 WT vs. HDAC6 KO). **f-g** BALB/c nude mice were implanted with 5 × 10⁶ non-SMC cells and subsequently treated with either the HDAC6 inhibitor Tubastatin A alone, CSF1R inhibitor BLZ945 alone, or a combination of both drugs. **h-i** Comparison of tumor weight and volume among different groups. **j** The body weight measurements of mice were taken every 3 days. **k** Metastatic burden comparison by tail vein injection of luciferase-labeled non-SMC cells. **l-m** Longitudinal monitoring via IVIS imaging. *P < 0.05; **P < 0.01; ***P < 0.001; ****P < 0.0001

Initially, we observed that HDAC6 in SCLC exhibited not only significant co-expression with EMT-related genes but also a strong negative correlation with multiple immune checkpoints (Fig. 2d), raising the possibility that HDAC6 contributes to the immunosuppressive tumor microenvironment (TME) in SCLC. Consistently, HDAC6 expression was negatively correlated with ESTIMATE, Stromal, and Immune scores in SCLC (Supplementary Fig. 5 d). However, the precise mechanisms by which HDAC6 regulates the SCLC immune TME remained unclear. Based on data from George et al*.*, we found that macrophages constitute the predominant immune cell population in SCLC (Supplementary Fig. 5e), and HDAC6 expression was inversely correlated with M1 macrophage-associated markers such as CD86 (Supplementary Fig. 5f). Together with our prior findings that HDAC6 modulates macrophage polarization in NSCLC [12], these results suggested that HDAC6 might contribute to the immunosuppressive TME in SCLC. To test this hypothesis, we first differentiated THP-1 cells into M0 macrophages with PMA treatment. Subsequently, these macrophages were co-cultured with both NCI-H1688 and SBC-2 cell lines in their HDAC6 WT and KO variants. To elucidate the underlying molecular changes, we extracted total mRNA from the co-cultured macrophages and conducted qRT-PCR experiments. Our results revealed that M1 macrophage-associated gene markers, such as CD86, CXCL10 and NOS2, were significantly up-regulated in macrophages co-cultured with HDAC6 KO NCI-H1688 and SBC-2 cell lines. Conversely, M2 macrophage-associated gene markers, including CD163, CD206, ARG1, and IL-10, were significantly down-regulated (Fig. 7a). To functionally validate these results, we performed transwell migration assays using conditioned media from WT or HDAC6 KO NCI-H1688/SBC-2 cells in the lower chamber, and observed the migration ability of macrophages in the upper chamber. Our results demonstrated that the migration ability of macrophages in the HDAC6 KO group was significantly enhanced (Fig. 7b). Collectively, these results demonstrated that HDAC6 regulated the M1/M2 polarization balance and suppresses macrophage recruitment, thereby contributing to the establishment of an immunosuppressive TME in SCLC.

In terms of clinical translation, currently, the efficacy of inhibitors targeting HDAC6 in solid tumors remained unsatisfactory. Based on our preliminary sequencing data, we have conducted further mining and analysis. Following HDAC6 KO, we identified numerous upregulated genes, and through intersectional analysis of RNA-seq and CUT&TAG data with |log2FC| and p-value filtering, we pinpointed CSF1R as a key candidate (Fig. 7c). As CSF1R was a critical regulator of M2 macrophage polarization, its upregulation upon HDAC6 KO might represent an important factor compromising the therapeutic efficacy of HDAC6 inhibitors. This finding was further validated in SCLC cell lines by RT-qPCR, confirming significant CSF1R upregulation after HDAC6 KO (Supplementary Fig. 5 g). Additionally, CUT&TAG sequencing analysis in HDAC6 KO cells demonstrated enhanced H3K27ac peaks near the TSS of the CSF1R gene (Fig. 7d). The Ch-IP assay demonstrated significantly reduced binding affinity of HDAC6 at H3K27ac-marked sites on the CSF1R promoter in HDAC6 KO NCI-H1688 cells compared to WT controls (Fig. 7e). These results collectively indicate that HDAC6 deficiency-induced CSF1R overexpression may contribute to treatment resistance by promoting an immunosuppressive M2 phenotype, highlighting the need for combination strategies targeting both HDAC6 and CSF1R pathways.

**Fig. 8:**
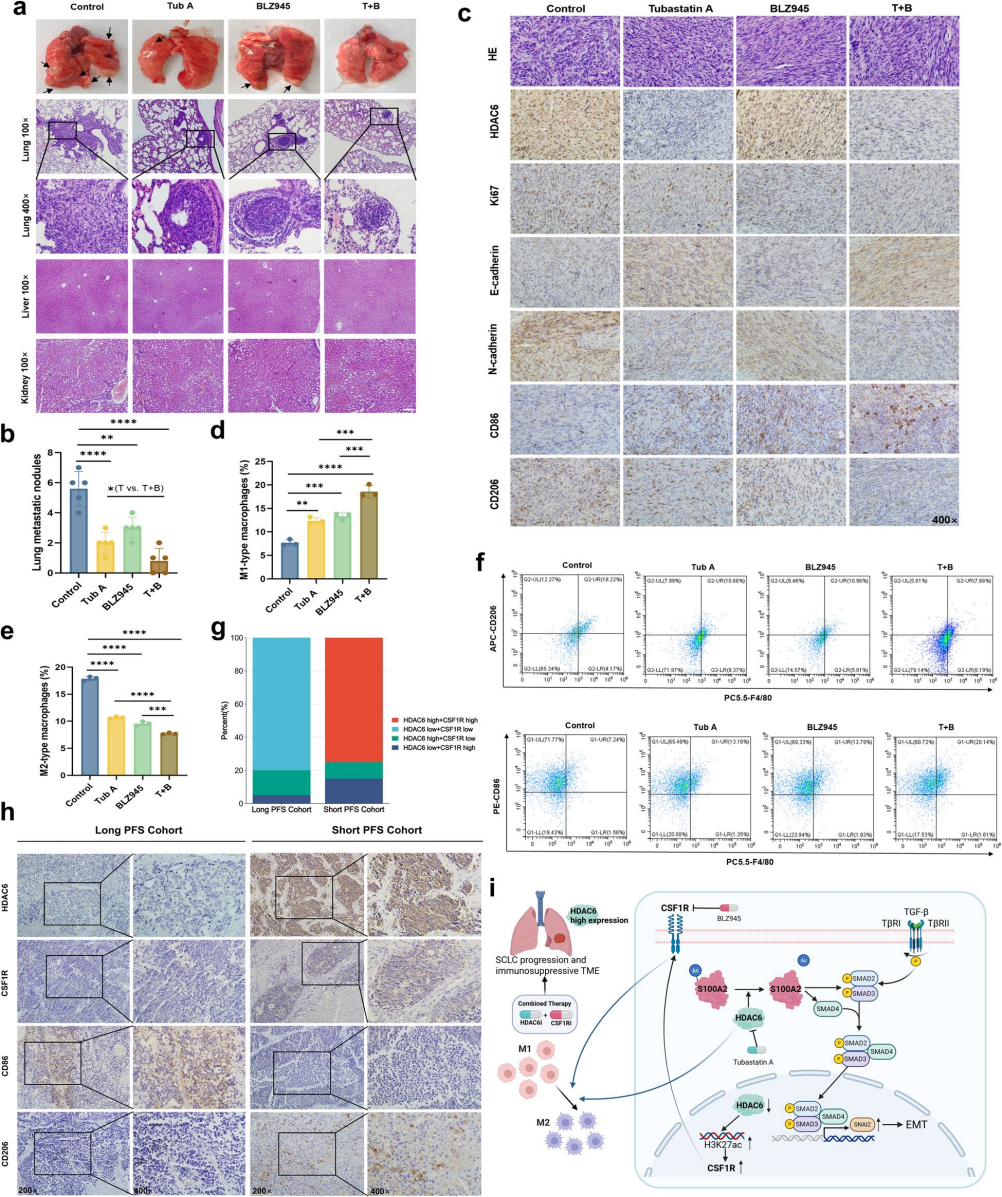
Dual HDAC6-CSF1R blockade synergistically shifted macrophages polarization toward an anti-tumor M1 phenotype in SCLC.** a-b** Comparison of pulmonary metastatic nodule counts (**a-b**), pulmonary metastatic lesion areas, liver and kidney by HE staining (**a**) across experimental groups. **c** Comparative IHC staining results of HDAC6, Ki67, E-Cadherin, N-Cadherin, CD86, and CD206 across experimental groups. **d-f** Flow cytometry analysis of M1/M2 macrophage proportions in tumor tissues across experimental groups. **g-h** IHC staining (**h**) and statistical results (**g**) of HDAC6, CSF1R, CD86, and CD206 in samples from the center with long and short PFS groups. **i** Mechanistic insights into HDAC6-mediated malignant progression and immune remodeling in SCLC. *P < 0.05; **P < 0.01; ***P < 0.001; ****P < 0.0001

Based on the preliminary experimental results, we conducted further exploration in animal models. Firstly, we injected non-SMC cells subcutaneously into BALB/C mice and divided them into four groups: a control group, an HDAC6 inhibitor monotherapy group (Tubastatin A), a CSF1R inhibitor BLZ945 monotherapy group, and a combination therapy group (Fig. 7f). We found that the combination therapy group produced the strongest suppression of tumor growth compared to either monotherapy groups, suggesting that HDAC6 inhibitors can synergize with CSF1R inhibitors to exert anti-tumor effects in SCLC (Fig. 7g-i). There was no significant difference in body weight among the four groups of mice (Fig. 7j). To further evaluate whether these drugs could suppress SCLC metastasis, we intravenously injected luciferase-labeled non-SMC cells into BALB/c mice via the tail vein, maintaining the same treatment groups and administration protocols as described previously (Fig. 7k). The results demonstrated that the HDAC6 inhibitor monotherapy group exhibited reduced metastatic burden, and the combination therapy group showed even more pronounced inhibition of metastasis (Fig. 7l-m), as evidenced by both histological assessment and visible metastatic nodule counts on lung surfaces (Fig. 8a-b). Importantly, HE staining showed no detectable pathological alterations in liver or kidney architecture across any treatment groups, suggesting favorable safety profiles for both individual compounds and their combination at the administered doses. Furthermore, IHC analysis of tumors from four groups revealed that the HDAC6 inhibitor-treated mice exhibited decreased Ki67 positivity, increased E-cadherin, and reduced N-cadherin, (Fig. 8c), suggesting inhibition of proliferation and EMT. By contrast, CSF1R inhibitor monotherapy produced minimal changes in these markers compared to controls, implying that its primary role is to modulate macrophage-dependent immunosuppression rather than directly interfering with EMT. Collectively, these findings suggest that HDAC6 inhibition suppresses SCLC progression partly through EMT regulation, whereas compensatory CSF1R upregulation sustains immunosuppression. Their combined blockade produces synergistic suppression of tumor growth and metastasis, providing a rationale for dual-targeting strategies in SCLC.

### Dual HDAC6-CSF1R blockade synergistically shifted macrophages polarization toward an anti-tumor M1 phenotype in SCLC.

Based on the established role of CSF1R in regulating macrophage polarization, we hypothesized that the combined efficacy of HDAC6 and CSF1R inhibitors might be mediated through synergistic modulation of macrophage polarization. To test this, we performed additional IHC staining for CD86 (M1 marker) and CD206 (M2 marker) across all four treatment groups. Our results revealed that both monotherapies increased CD86-positive macrophage infiltration, whereas combination therapy further enhanced M1 polarization. Conversely, CD206 expression exhibited the opposite trend. To complement these findings, we conducted flow cytometry on single-cell suspensions prepared from dissociated tumor tissues. We observed that both Tubastatin A (HDAC6i) and BLZ945 (CSF1Ri) monotherapies significantly promoted M1 macrophage polarization within the SCLC TME. Notably, the combination therapy group showed even more substantial increases in M1 populations, with corresponding decreases in M2 macrophages, suggesting synergistic reprogramming of the immunosuppressive TME (Fig. 8d-f). Collectively, these data partially support our original postulate that the two agents cooperatively modulate macrophage polarization in vivo.

To further explore the impact of HDAC6 and CSF1R on macrophage polarization and patient survival outcomes in clinical samples, we performed IHC staining for HDAC6, CSF1R, CD86 (M1 marker), and CD206 (M2 marker) in 40 cases from our institutional cohort, including 20 cases with long PFS and 20 with short PFS. The results demonstrated that 80% of the long PFS group exhibited dual low expression of HDAC6 and CSFF1R with higher CD86-positive cell infiltration, while 75% of the short PFS group showed dual high expression of HDAC6 and CSF1R with predominant CD206-positive cells (Fig. 8g-h). Building upon these clinical findings and our previous experimental results, we propose that HDAC6 and CSF1R may serve as promising dual therapeutic targets in SCLC, with their combined inhibition potentially reprogramming the TME and improving clinical outcomes for SCLC patients.

## Discussion

HDAC6 is distinguished from other histone deacetylases due to its unique cytoplasmic localization and ability to target non-histone substrates [28], thereby regulating critical cancer hallmarks such as protein stability [29], cellular motility [30], and immune modulation [31]. In this study, we identified HDAC6 as consistently overexpressed in SCLC, particularly within ASCL1^+^ and NEUROD1^+^ subtypes, where elevated expression correlated with advanced disease stage and inferior survival. Beyond its established cellular functions, our work reveals that HDAC6 orchestrates metastatic and immunosuppressive programs in SCLC through two interconnected signaling axes: S100A2-TGF-β/SMAD and CSF1R. These findings underscore HDAC6 as a clinically relevant driver of tumor aggressiveness in SCLC, a disease in which therapeutic innovation beyond chemotherapy and immunotherapy has been limited.

Through integrated multi-omics analyses and functional validation, we revealed that HDAC6 as a key driver of SCLC malignancy via two interconnected mechanisms (Fig. 8i). First, HDAC6 activates the TGF-β/SMAD pathway through deacetylation of the novel substrate S100A2. Specifically, HDAC6 directly interacts with S100A2 via its second deacetylase domain and mediates deacetylation at K27, stabilizing the SMAD2/3/4 complex and enhancing its nuclear translocation. This modification facilitated transcriptional activation of EMT drivers, including SNAI2. Acetylation-mimicking or deacetylation-mimicking mutants of S100A2 confirmed the causal link between S100A2 acetylation status and downstream pathway activation. While S100A2 has been reported to play context-dependent roles in other malignancies—including oncogenic roles in NSCLC [32], pancreatic [26], colorectal [33, 34], and renal cancers [35], but tumor-suppressive effects in hepatocellular carcinoma [36]—our results establish its oncogenic activity in SCLC as HDAC6-dependent, expanding the functional repertoire of HDAC6 substrates beyond structural proteins to include signaling adaptors. Our findings are further supported by a growing body of evidence demonstrating that HDAC6 regulates non-canonical pathways. For instance, multiple screening approaches have revealed HDAC6 as a novel regulator of glycolytic metabolism in triple-negative breast cancer, identifying a cohort of non-traditional, metabolism-associated substrates [37]. Together with our functional validation of S100A2 in lung cancer, this evidence underscores the paradigm that unbiased screening strategies are powerful tools for uncovering a broader and more diverse substrate spectrum for HDAC6, moving far beyond its traditional roles. While, HDAC6's broad substrate specificity and central role in multiple cellular processes also present a key challenge, as off-target effects could narrow the therapeutic window. Advancing this strategy necessitates developing more selective HDAC6 inhibitors and identifying predictive biomarkers for precise patient stratification.

Second, HDAC6 regulates the tumor immune microenvironment by promoting macrophage polarization toward the M2 phenotype. HDAC6 deficiency enhanced secretion of CXCL9/10, favoring an M1-like state, and synergized with CSF1R inhibition to reverse immunosuppression. Importantly, we observed compensatory CSF1R upregulation upon HDAC6 inhibition, mediated by H3K27ac-dependent chromatin remodeling, suggesting a feedback loop that may underlie the limited efficacy of HDAC6 monotherapy. Our findings align with established mechanisms demonstrating HDAC6's role in mediating transcriptional reprogramming through H3K27ac modulation. Previous studies have reported that HDAC6 inactivation enhances p300 stability and increases H3K27ac levels [38]. Additionally, research in colorectal cancer has shown that β-catenin employs HDAC6-mediated H3K27 histone modification to transcriptionally repress lnc-MIR100HG expression [39]. These documented pathways directly support our model wherein HDAC6 inhibition leads to H3K27ac alteration and subsequent transcriptional reprogramming, providing independent validation for our proposed mechanism. In T cell–deficient models, combined inhibition of HDAC6 and CSF1R effectively reprogrammed macrophages and significantly suppressed tumor growth, providing preclinical validation for this rational therapeutic combination. These findings are consistent with recent single-cell transcriptomic studies that identified CSF1R⁺ monocytic cells as mediators of immunotherapy resistance—specifically through suppression of CAR-T cell function via the PGE2-EP2/EP4 axis [40]. This collective evidence reinforces the emerging concept of CSF1R as an immune checkpoint-like regulator with broad relevance in oncology. The CSF1R inhibitor vimseltinib has completed Phase I clinical trials in patients with advanced solid tumors or tenosynovial giant cell tumor, demonstrating favorable long-term tolerability, manageable safety profile, and significant antitumor activity [41]. These findings provide clinical evidence supporting CSF1R as a promising therapeutic target for cancer treatment. However, some clinical trials have shown disappointing results with CSF1R inhibitor monotherapy in malignant solid tumors. It has been suggested that the lack of efficacy may be partly attributable to the requirement of active engagement from the adaptive immune system for long-term tumor control, which may not be sufficiently triggered by TAM-targeted approaches alone [42]. Therefore, the clinical application of CSF1R inhibitors remains challenging. Although the dual inhibition of HDAC6 and CSF1R demonstrates significant synergistic anti-tumor efficacy in our study, its clinical translation requires careful consideration. This potent combination, while suppressing tumor escape mechanisms, may itself impose substantial evolutionary pressure that could potentially trigger unforeseen resistance through alternative pathway activation (e.g., PI3K/AKT or WNT) or epigenetic reprogramming [43–45]. Consequently, future studies on this combination therapy must include close monitoring of patient samples to facilitate early identification of emerging resistance mechanisms.

Beyond these mechanistic insights, our findings underscore the clinical implications of HDAC6 in SCLC metastasis. SCLC exhibits a high propensity for brain metastasis [2]. For SCLC cells to successfully colonize the brain, they must traverse the protective BBB. The BBB, composed of brain microvascular endothelial cells interconnected by tight junctions and other components, prevents most chemotherapeutic and targeted agents from effectively reaching intracranial lesions, posing a major therapeutic challenge [46]. Previous studies have reported that HDAC6 enhances cell motility, participates in the formation of invasive pseudopodia, facilitates proteolytic degradation of the extracellular matrix, and promotes tumor cell survival by enabling anchorage-independent proliferation—all critical steps in the metastatic cascade [19]. This suggests that HDAC6 may play an important role in facilitating metastasis. Our findings align with these observations: HDAC6 knockout significantly reduced metastatic burden in vivo, and an in vitro BBB model confirmed its crucial role in promoting tumor cell penetration. Furthermore, analysis of patient samples revealed co-localization of HDAC6 with the metastasis-associated marker MMP9. Collectively, these discoveries provide a rationale for developing BBB-penetrant HDAC6 inhibitors as a novel therapeutic strategy to prevent or treat brain metastases, which holds significant promise for addressing this major clinical challenge in SCLC.

This study has limitations. While our data support a central role for the HDAC6-S100A2-TGF-β/SMAD axis and HDAC6-driven immune modulation, additional substrates and pathways may contribute to the observed phenotypes. Furthermore, due to the incompatibility of the non-SMC cell line with immunocompetent C57BL/6 mice, the in vivo experiments in this study were primarily conducted in immunodeficient models. Consequently, while our findings elucidate the impact of HDAC6 on innate immunity—specifically macrophage polarization—this experimental constraint limits a comprehensive interpretation of the complete tumor immune microenvironment. Larger clinical cohorts and functional studies in immune-competent models will be required to further validate these mechanisms and their translational potential.

In summary, this study established HDAC6 as a critical nexus connecting epigenetic regulation, metastatic signaling, and immune evasion in SCLC. These findings suggest two translational directions: (i) biomarker-driven identification of patients with HDAC6-high tumors, particularly ASCL1/NEUROD1 subtypes, and (ii) clinical evaluation of combination regimens pairing selective HDAC6 inhibitors (e.g., ACY-1215) with CSF1R blockade. Such dual-targeting strategy strategies may simultaneously suppress tumor growth, prevent brain metastases, and reprogram the immunosuppressive microenvironment, offering a promising therapeutic avenue for one of the most lethal human cancers.

## Supplementary Information

Supplementary Material 1.

## Data Availability

All data supporting the findings of this study are presented in the article and the Supplementary Materials. All other raw data are available upon reasonable request from the corresponding author.

## Abbreviations

ATCC: American Type Culture Collection
BBB: Blood-Brain Barrier
Ch-IP: Chromatin immunoprecipitation
CDs: Catalytic domains
EMT: Epithelial-mesenchymal transition
ES-SCLC: Extensive-stage SCLC
HDAC6: Histone deacetylase 6
HAT: Histone acetyltransferases
ICIs: Immune checkpoint inhibitors
IHC: Immunohistochemistry
IF: Immunofluorescent
KO: Knockout
mOS: Median overall survival
NSCLC: Non-small cell lung cancer
OS: Overall survival
OE: Overexpression
PFS: Progression-free survival
SCLC: Small cell lung cancer
Wb: Western blotting
