# Supplementary material for: HDAC6 orchestrates metastatic and immunosuppressive programs in small cell lung cancer through S100A2-TGF-β/SMAD and CSF1R signaling
Source: Mol Cancer. 2026 Jan 7;25:33. doi: 10.1186/s12943-025-02552-y (PMC12908322; doi:10.1186/s12943-025-02552-y)
Supplement: Supplementary file 1 — Supplementary Material 1. [file 12943_2025_2552_MOESM1_ESM.docx]

**Supplementary Material**

**HDAC6 orchestrates metastatic and immunosuppressive programs in small cell lung cancer through S100A2-TGF-β/SMAD and CSF1R signaling**

Yantao Jiang^1^, Junjie Yu^1^, Ting Wang^1^, Qingwu Du^1^, Jingya Wang^1^, Yi Lu^1^, Qi Xu^1^, Huiyan Liu^1^, Xueyang Li^1^, Luyao Tong^1^, Tingting Qin^1*^, and Dingzhi Huang^1*^

**Supplementary Figures**

**Supplementary Figure 1**

**Supplementary Figure 1.** **HDAC6 promoted the** **proliferation and aggressiveness of SCLC cells.** **a** The mRNA expression of HDAC6 across tissue types in GSE116977 dataset. **b** The mRNA expression of HDAC6 in SCLC with BM and SCLC without BM tissues in GSE161968 dataset. **c** WB and qRT-PCR validated HDAC6 OE efficiency at protein and mRNA levels. **d-e** CCK8 (**d**) and clone formation assay (**e**) between WT and HDAC6 OE cell lines. **f** WB experiments of EMT-related key molecules in WT and HDAC6 OE cell lines. **g-h** Transwell (**g**) and wound healing assay (**h**) in WT and HDAC6 OE cell lines. **i** WB and qRT-PCR validated HDAC6 OE rescue efficiency at protein and mRNA levels. **j-m** CCK8 (**j**) and clone formation assay (**k**), Transwell assay (**l**) and wound healing assay (**n**) in HDAC6 KO and HDAC6 KO+OE cell lines. **p* < 0.05; ***p* < 0.01; ****p* < 0.001; *****p*< 0.0001

**Supplementary Figure 2**

**

**

**Supplementary Figure 2.** **HDAC6 inhibitor Tubastatin A suppressed malignant progression in SCLC.** **a** WB experiments of EMT-related key molecules in HDAC6 KO and KO+OE cell lines. **b** The 24 h and 48 h IC50 values of Tubastatin A in SBC-2 and H1688 cell lines. **c-d** CCK8 (**c**) and clone formation assay (**d**) between control and drug-treated cell lines. **e-g** WB and qRT-PCR experiments of apoptotic **(e-f**) and EMT (**g**) pathway-related key molecules in control and drug-treated cell lines. **h-j** Transwell assay (**h-i**) and wound healing assay (**j**) in control and drug-treated cell lines. **k** HBMEC/NHA co-culture model of the BBB. **l-m** BBB permeability tests: 4h leakage (**l**) and fluorescein sodium penetration assays (**m**). **n-o** The protein (**n**) and mRNA (**o**) changes of tight junctions across BBB co-culture phases. **p* < 0.05; ***p* < 0.01; ****p* < 0.001; *****p*< 0.0001

**Supplementary Figure 3**

**

**

**Supplementary Figure 3.** **a-b** Longitudinal monitoring between the two groups was performed via IVIS imaging following intracardiac injection. **c** Volcano plot of differentially expressed genes between NCI-H1688 HDAC6 WT and KO groups from mRNA-seq. **d** Pie chart of functional subcellular localization for differential DPs. **e** Heatmap of EMT-related markers from mRNA-seq data. **f** Volcano plot of differentially expressed transcription factors from mRNA-seq data. **g-h** Functional enrichment analysis of differential genes by GO and KEGG based on mRNA-seq data (**g**) and CUT&TAG data (**h**), respectively.

**Supplementary Figure 4**

**

Supplementary Figure 4.** **a** Heatmap of differentially acetylated proteins between groups. **b** HDAC6-mediated deacetylation sites. **c-d** GO (**c**) and KEGG (**d**) pathway enrichment analysis. **e** Differences in S100A2 expression levels between tumor and adjacent normal tissues across various cancer types based on TCGA database. **f** Impact of S100A2 expression on OS and PFS across multiple cancer types based on GEPIA2.0 database. **g** WB and qRT-PCR validated S100A2 OE efficiency at protein and mRNA levels, respectively. **h** WB and qRT-PCR validated S100A2 OE efficiency at protein and mRNA levels in HDAC6 KO cells, respectively. **p* < 0.05; ***p* < 0.01; ****p* < 0.001; *****p*< 0.0001

**Supplementary Figure 5**

**

**

**Supplementary Figure 5. a-b** WB and qRT-PCR validated SMAD3 KO efficiency at protein and mRNA levels, respectively in H1688 (**a**) and SBC-2 (**b**) cell lines. **c** WB experiments of EMT markers in WT and SMAD3 KO cell lines. **d** Correlations between HDAC6 expression and Estimate score, Stroma score and Immune score. **e** Immune cell composition in SCLC tumor microenvironment (George et al.). **f** The correlation between HDAC6 and the expression of M1 macrophage-related markers. **g** The difference in CSF1R mRNA expression between HDAC6 WT and KO groups in H1688 and SBC-2 cell lines.

**Supplementary Tables**

**Supplementary Table 1**

| **Characteristics** | **Total** | **HDAC6-Low** | **HDAC6-High** | **P** |
| --- | --- | --- | --- | --- |
|  | **N=98 (%)** | **N=42 (%)** | **N=56 (%)** |  |
| \| Age \| \| --- \| \| Mean±SD \| \| Median (min-max) \| | 61.11±8.32  61 (35,77) | 59.60±9.17  59.5 (35,77) | 62.25±7.51  62 (44,76) | 0.40 |
| Gender  Female  Male | 16 (16.33%)  82 (83.67%) | 9 (21.43%)  33 (78.57%) | 7 (12.5%)  49 (87.5%) | 0.36 |
| Smoking history  NO  YES | 13 (13.27%)  85 (86.73%) | 6 (14.29%)  36 (85.71%) | 7 (12.5%)  49 (87.5%) | 0.87 |
| ECOG  0  1  2 | 62 (63.27%)  29 (29.59%)  7 (7.14%) | 27 (64.29%)  12 (28.57%)  3 (7.14%) | 35 (62.5%)  17 (30.36%)  4 (7.14%) | 0.98 |
| T-Stage  I  II  III  IV | 55 (56.12%)  27 (27.55%)  10 (10.20%)  6 (6.12%) | 25 (59.52%)  12 (28.57%)  3 (7.14%)  2 (4.76%) | 30 (53.57%)  15 (26.79%)  7 (12.5%)  4 (7.14%) | 0.78 |
| N-Stage  0  1  2  3 | 57 (58.16%)  11 (11.22%)  29 (29.59%)  1 (1.02%) | 26 (61.9%)  2 (4.76%)  13 (30.95%)  1 (2.38%) | 31 (55.36%)  9 (16.07%)  16 (28.57%)  0 (0.00%) | 0.23 |
| M-Stage  0  1 | 83 (84.69%)  15 (15.31%) | 36 (85.71%)  6 (14.29%) | 47 (83.93%)  9 (16.07%) | 1.00 |
| pTNM-Stage  I  II  III  IV | 39 (39.80%)  16 (16.33%)  30 (30.61%)  13 (13.27%) | 17 (40.48%)  6 (14.29%)  14 (33.33%)  5 (11.90%) | 22 (39.29%)  10 (17.86%)  16 (28.57%)  8 (14.29%) | 0.92 |
| Progress Status  NO  YES | 31 (31.63%)  67 (68.37%) | 18 (42.86%)  24 (57.14%) | 13 (23.21%)  43 (76.79%) | 0.06 |
| OS Status  Live  Dead | 36 (36.73%)  62 (63.27%) | 22 (52.38%)  20 (47.62%) | 14 (25.00%)  42 (75.00%) | 0.01 |

**Supplementary Table 2**

| **Gene** | **Forward prime** | **Reverse prime** |
| --- | --- | --- |
| β-ACTIN | 5'-TGACGTGGACATCCGCAAAG-3' | 5’-CTGGAAGGTGGACAGCGAGG-3' |
| HDAC6 | 5'-GCCTCAATCACTGAGACCATCC-3' | 5'-GGTGCCTTCTTGGTGACCAACT-3' |
| S100A2 | 5'-TGCCAAGAGGGCGACAAGTTCA-3' | 5'-AAGTCCACCTGCTGGTCACTGT-3' |
| CSF1R | 5'-GCTGCCTTACAACGAGAAGTGG-3' | 5'-CATCCTCCTTGCCCAGACCAAA-3' |
| Bcl-2 | 5'-ATCGCCCTGTGGATGACTGAGT-3' | 5'-GCCAGGAGAAATCAAACAGAGGC-3' |
| Bax | 5'-TCAGGATGCGTCCACCAAGAAG-3' | 5'-TGTGTCCACGGCGGCAATCATC-3' |
| Bim | 5'-CAAGAGTTGCGGCGTATTGGAG-3' | 5'-ACACCAGGCGGACAATGTAACG-3' |
| CD86 | 5'-CCATCAGCTTGTCTGTTTCATTCC-3' | 5'-GCTGTAATCCAAGGAATGTGGTC-3' |
| CXCL9 | 5'-CTGTTCCTGCATCAGCACCAAC-3' | 5'-TGAACTCCATTCTTCAGTGTAGCA-3' |
| CXCL10 | 5'-GGTGAGAAGAGATGTCTGAATCC-3' | 5'-GTCCATCCTTGGAAGCACTGCA-3' |
| TNF-α | 5'-CTCTTCTGCCTGCTGCACTTTG-3' | 5'-ATGGGCTACAGGCTTGTCACTC-3' |
| NOS2 | 5'-GCTCTACACCTCCAATGTGACC-3' | 5'-CTGCCGAGATTTGAGCCTCATG-3' |
| CD163 | 5'-CCAGAAGGAACTTGTAGCCACAG-3' | 5'-CAGGCACCAAGCGTTTTGAGCT-3' |
| CD206 | 5'-AGCCAACACCAGCTCCTCAAGA-3' | 5'-CAAAACGCTCGCGCATTGTCCA-3' |
| ARG1 | 5'-TCATCTGGGTGGATGCTCACAC-3' | 5'-GAGAATCCTGGCACATCGGGAA-3' |
| IL-10 | 5'-TCTCCGAGATGCCTTCAGCAGA-3' | 5'-TCAGACAAGGCTTGGCAACCCA-3' |
| TGF-β | 5'-TACCTGAACCCGTGTTGCTCTC-3' | 5'-GTTGCTGAGGTATCGCCAGGAA-3' |
| ZO-1 | 5'-GTCCAGAATCTCGGAAAAGGTGCC-3' | 5'-CTTTCAGCGCACCATACCAACC-3' |
| Claudin-5 | 5'-ATGTGGCAGGTGACCGCCTTC-3' | 5'-CGAGTCGTACACTTTGCACTGC-3' |
| Occludins | 5'-ATGGCAAAGTGAATGACAAGCGG-3' | 5'-CTGTAACGAGGCTGCCTGAAGT-3' |
| SNAI2 P1 | 5'-GCTGGGACAGAGTCTTGACAG-3' | 5'-CCACAGGGTCCCTGAGTTTCT-3' |
| SNAI2 P2 | 5'-TCCCTGGGTGTGTCTGTGTCT-3' | 5'-AGGCACAGGGACAGGAAGAGA-3' |
| SNAI2 P3 | 5'-GGTGTGGCTGGGAAGTCCT-3' | 5'-CACACACCCACACACACACC-3' |
| CSF1R P1 | 5'-CTGGTGGGAGGTAAGGCAAG-3' | 5'-AGGTGGCTGGGAGAATCAAT |
| CSF1R P2 | 5'-AGGAGGATCGAGGGTAAAGG-3' | 5'-GCCAGGGACAGTGATTGAGA-3' |
| CSF1R P3 | 5'-AGTGAGAAGGGTGAGGAGTT-3' | 5'-TGCCCAGTCTTTTATAGCTG-3' |
| CSF1R P4 | 5'-AAGGGAACACCAAGACCATC-3' | 5'-GGAAGAAAATCTGCCACCAC-3' |

| **sgRNA Sequences** |  |
| --- | --- |
| sgHDAC6#1 | CCGCTCTATCCCCAATCTAG |
| sgHDAC6#2 | ACCTAATCGTGGGACTGCAA |
| sgHDAC6#3 | GAAAGGACACGCAGCGATCT |
| sgSMAD3#1 | TGCGGCTCTACTACATCGGA |
| sgSMAD3#2 | AATTCGGAGCGCTTCTGCCT |
| sgSMAD3#3 | GCCATAGCGCTGGTTACAGT |

**Supplementary Table 3**

**Uncropped images of the WB**

**Figure. 1i**

**
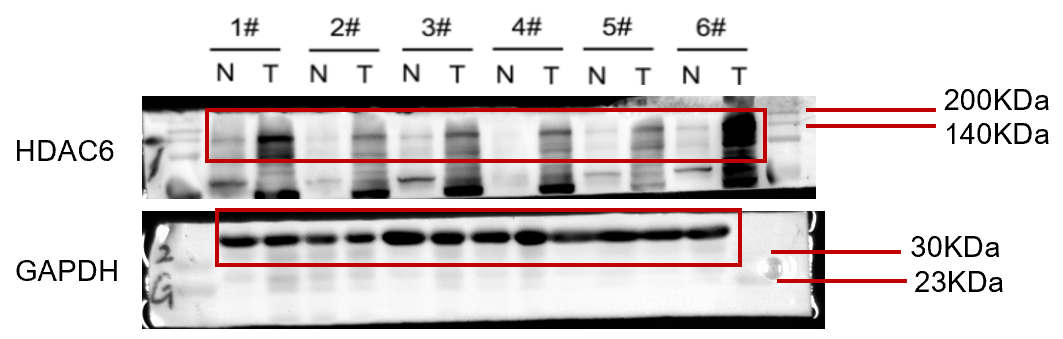
**

**Figure. 2e**

**
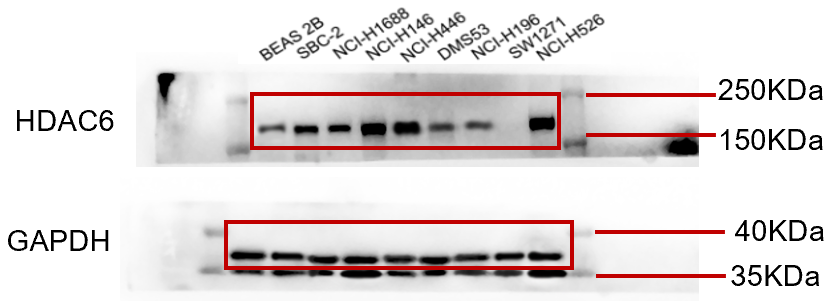
**

**Figure. 2f**

**
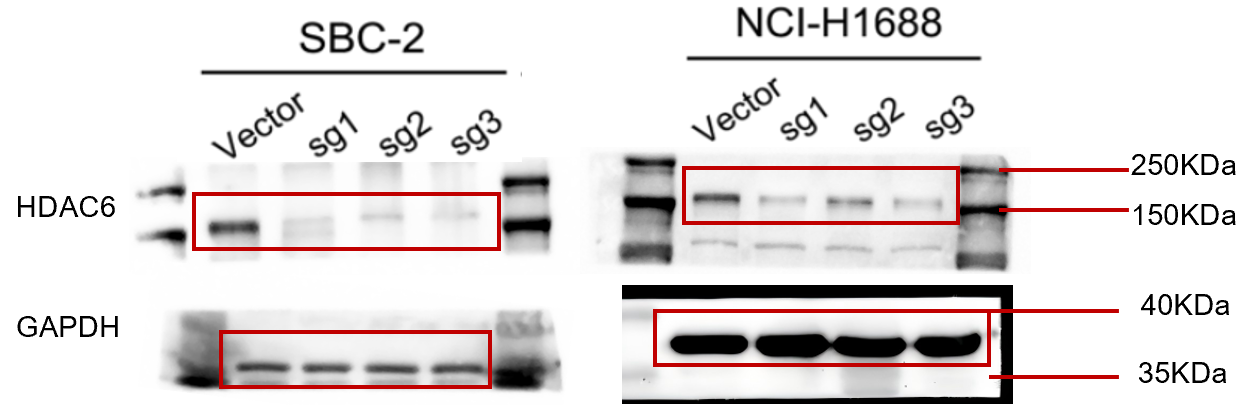
**

**Figure. 2f**

**
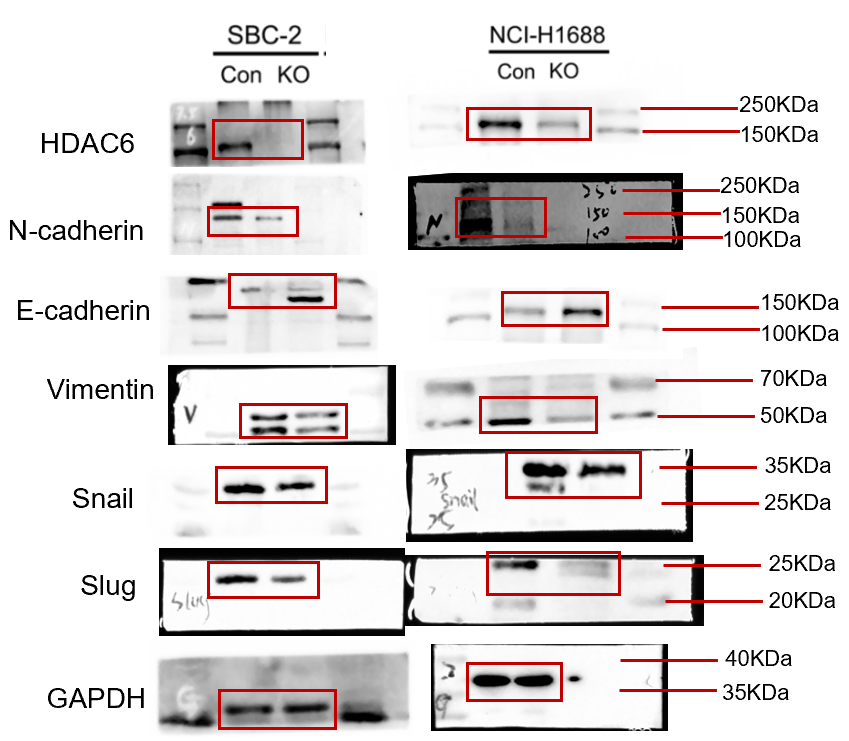
**

**Figure. 2i**

**
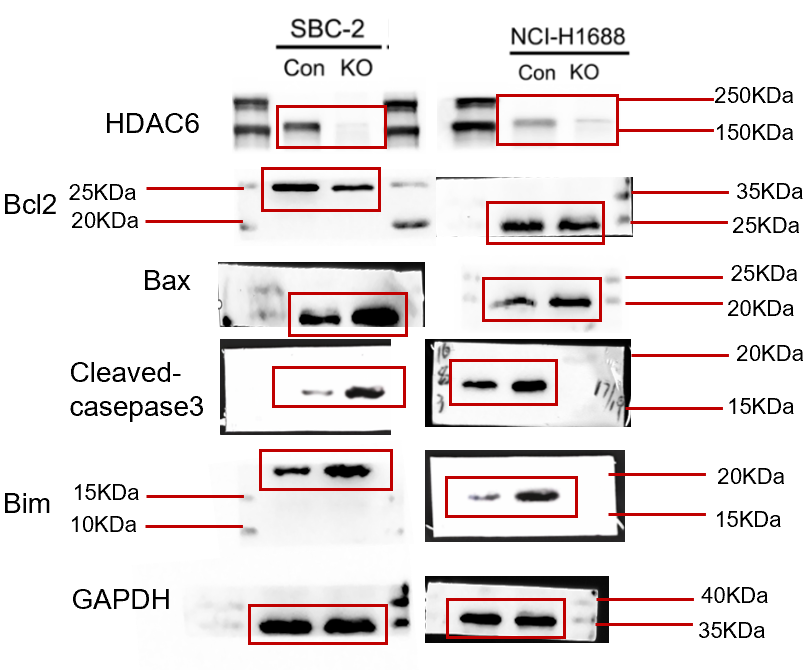
**

**Figure. 5i**

**
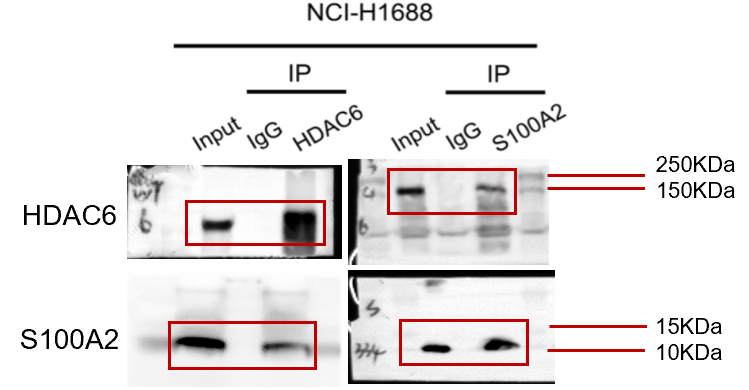

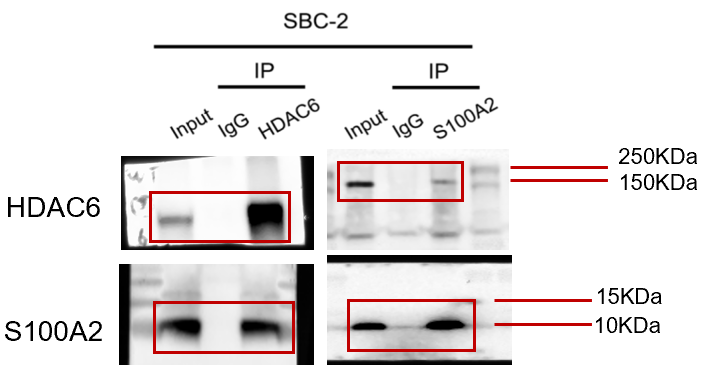
**

**Figure. 5j Figure. 5l**

**
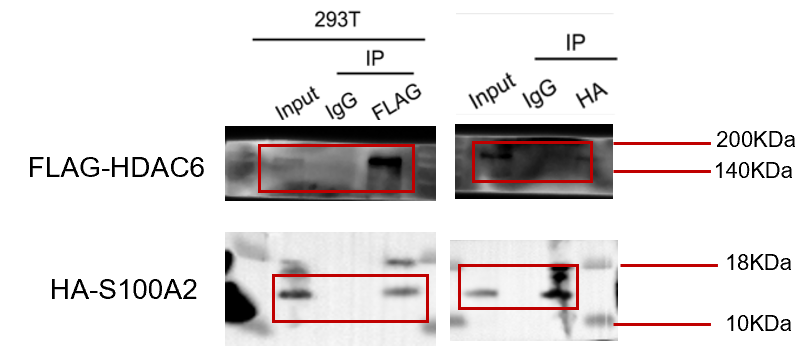

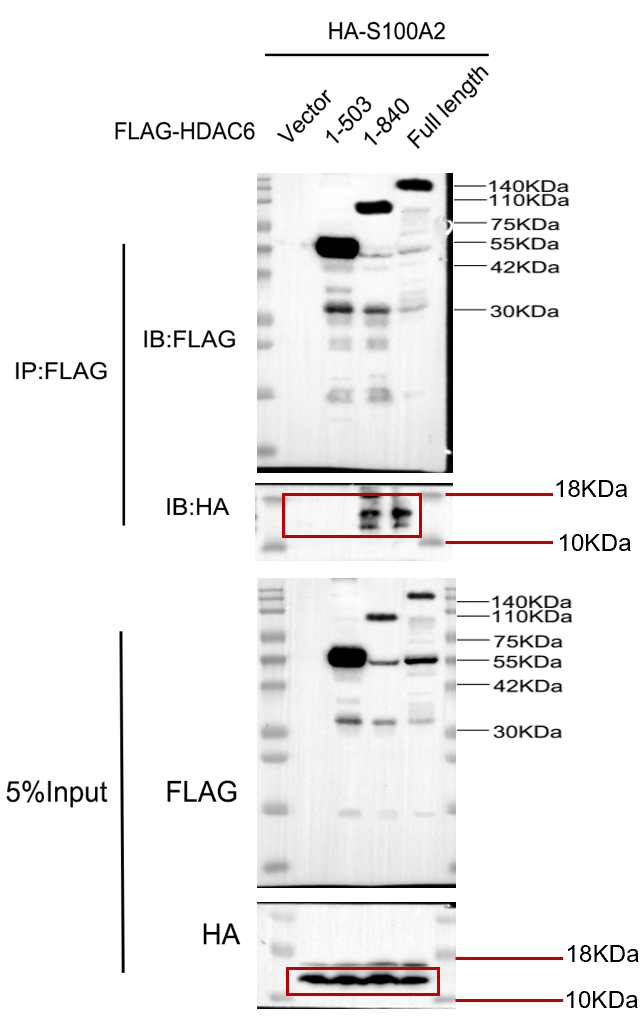
**

**Figure. 5m Figure. 5n**

**
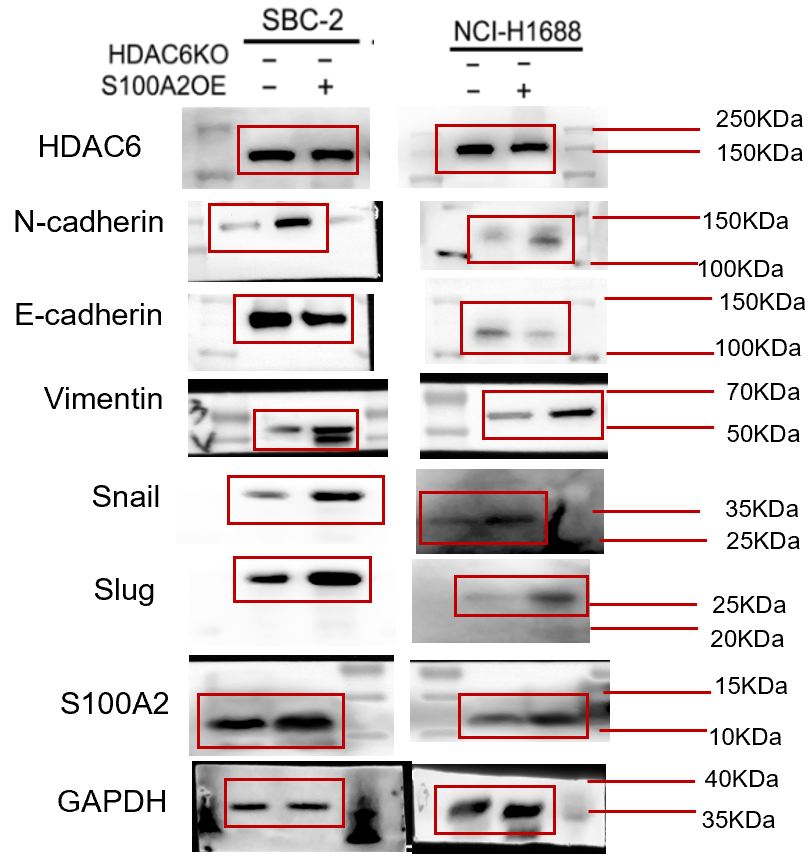

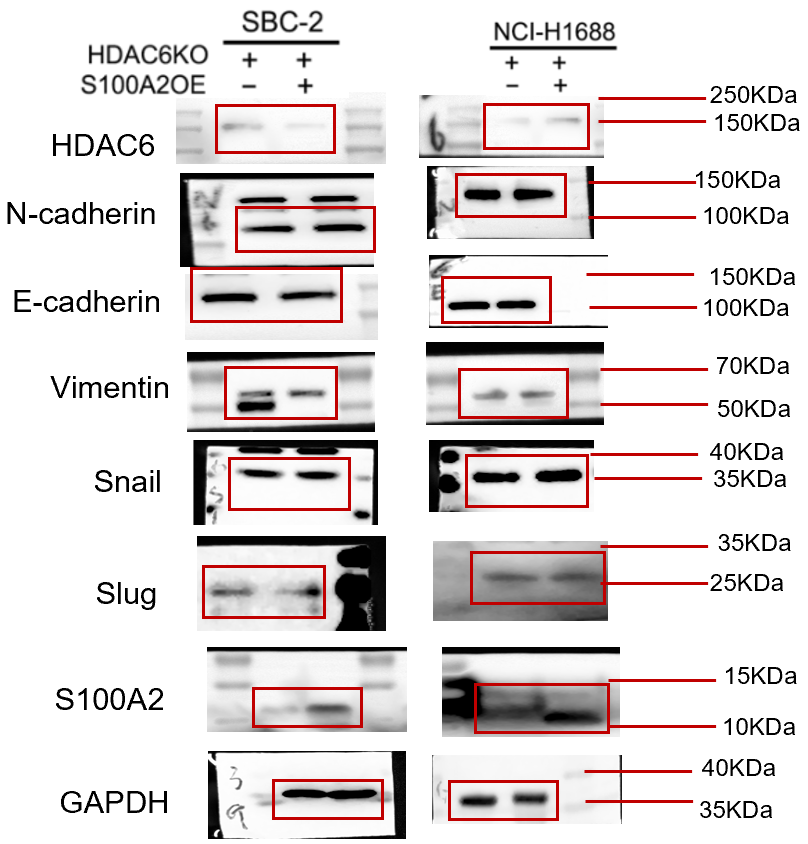
**

**Figure. 5o Figure. 5p**

**
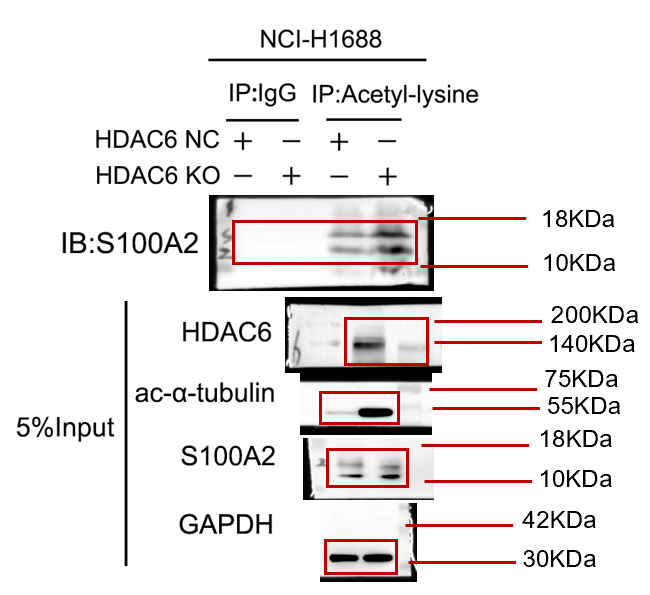
** **
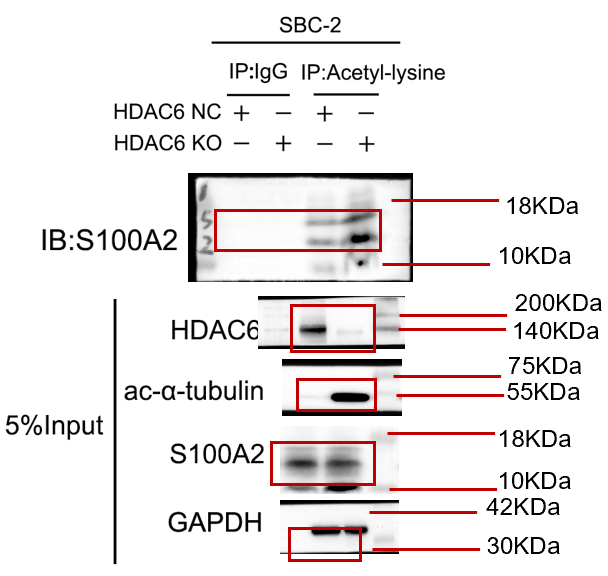
**

**Figure. 5q Figure. 5r**

**
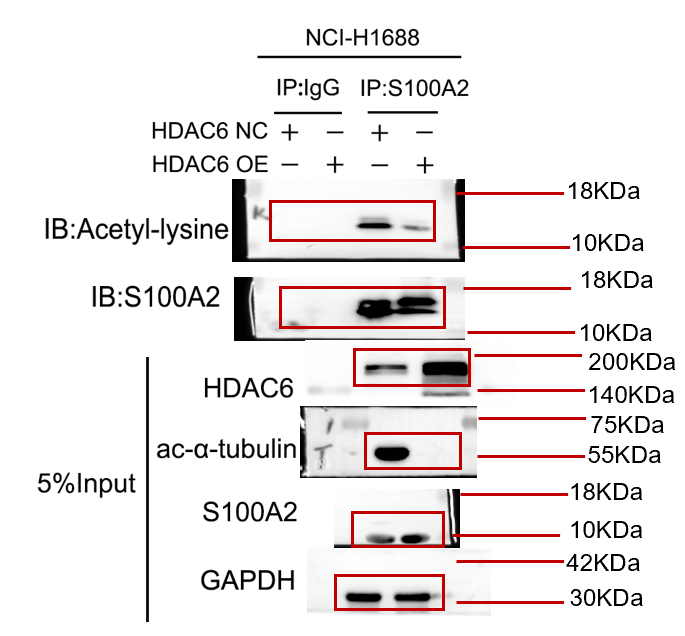
** **
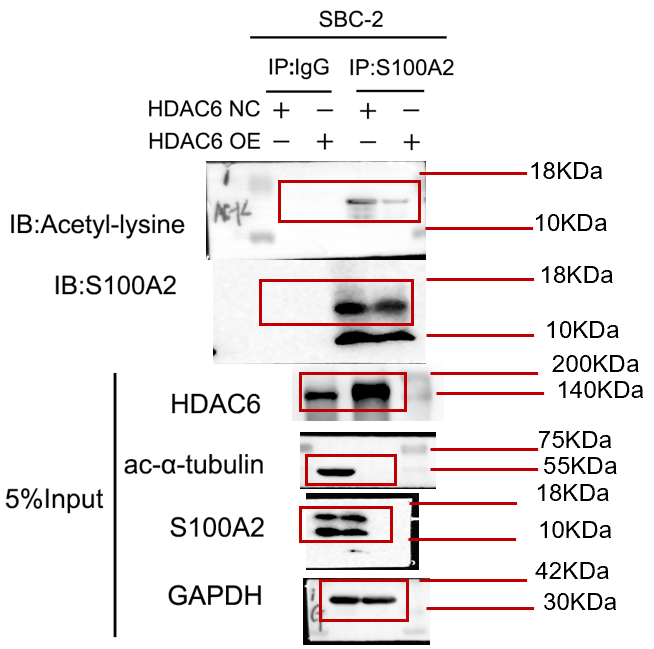
**

**Figure. 5s Figure. 5t**

**
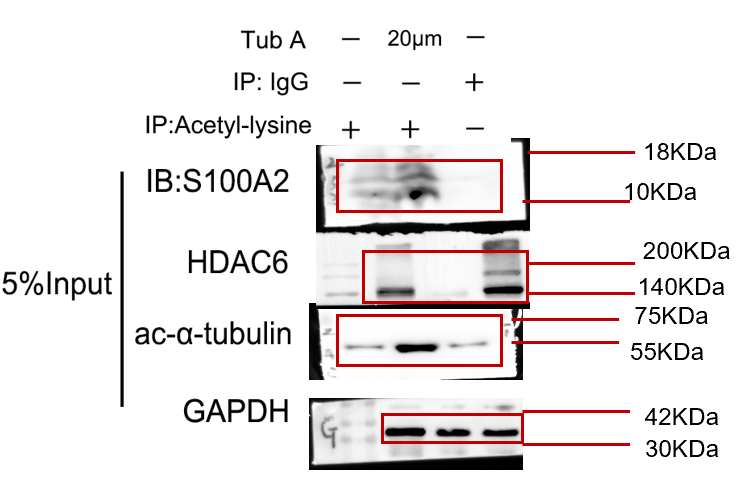
** **
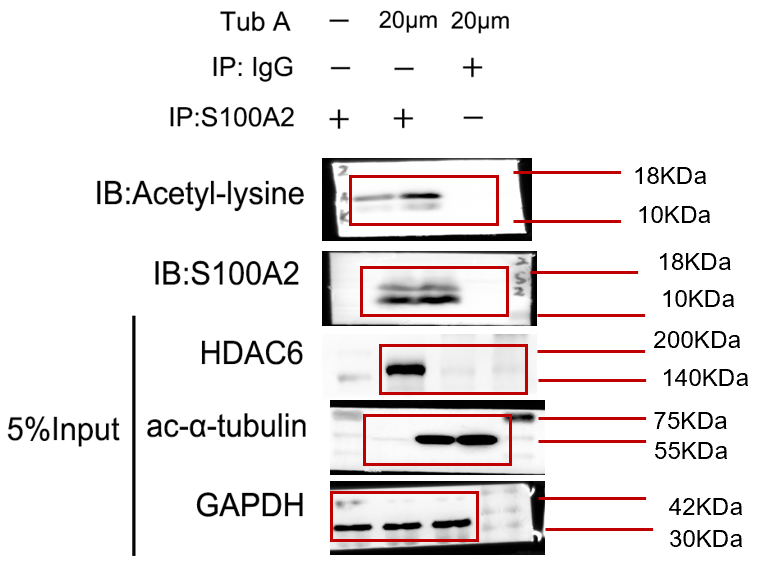
**

**Figure. 6c Figure. 6d**

**
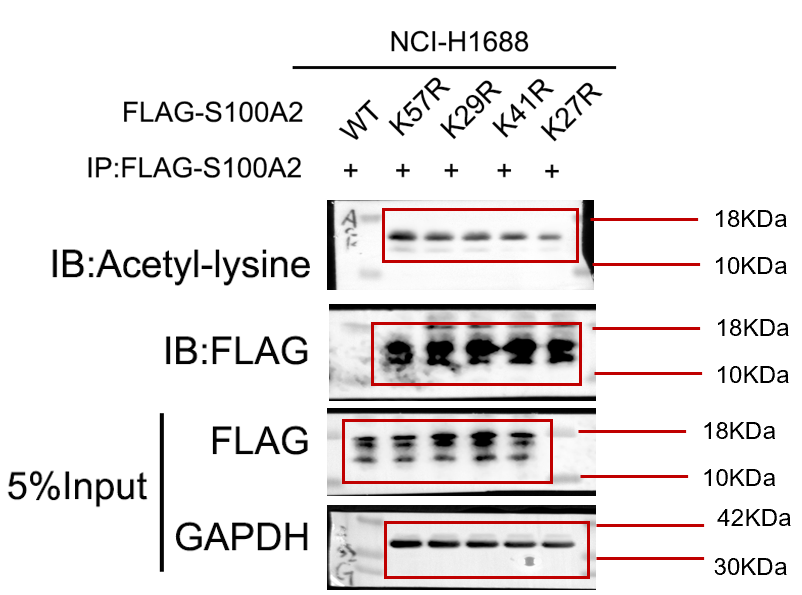
** **
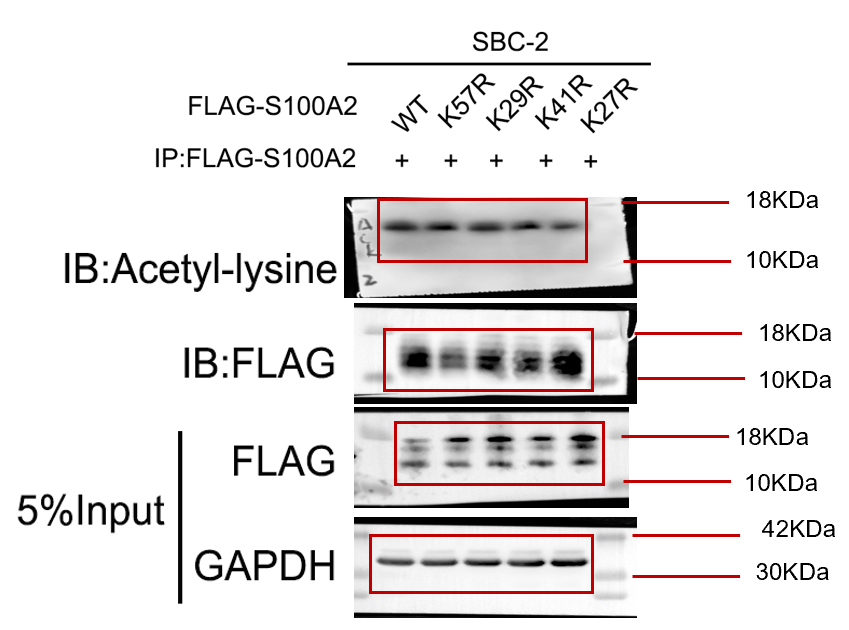
**

**Figure. 6e Figure. 6f**

**
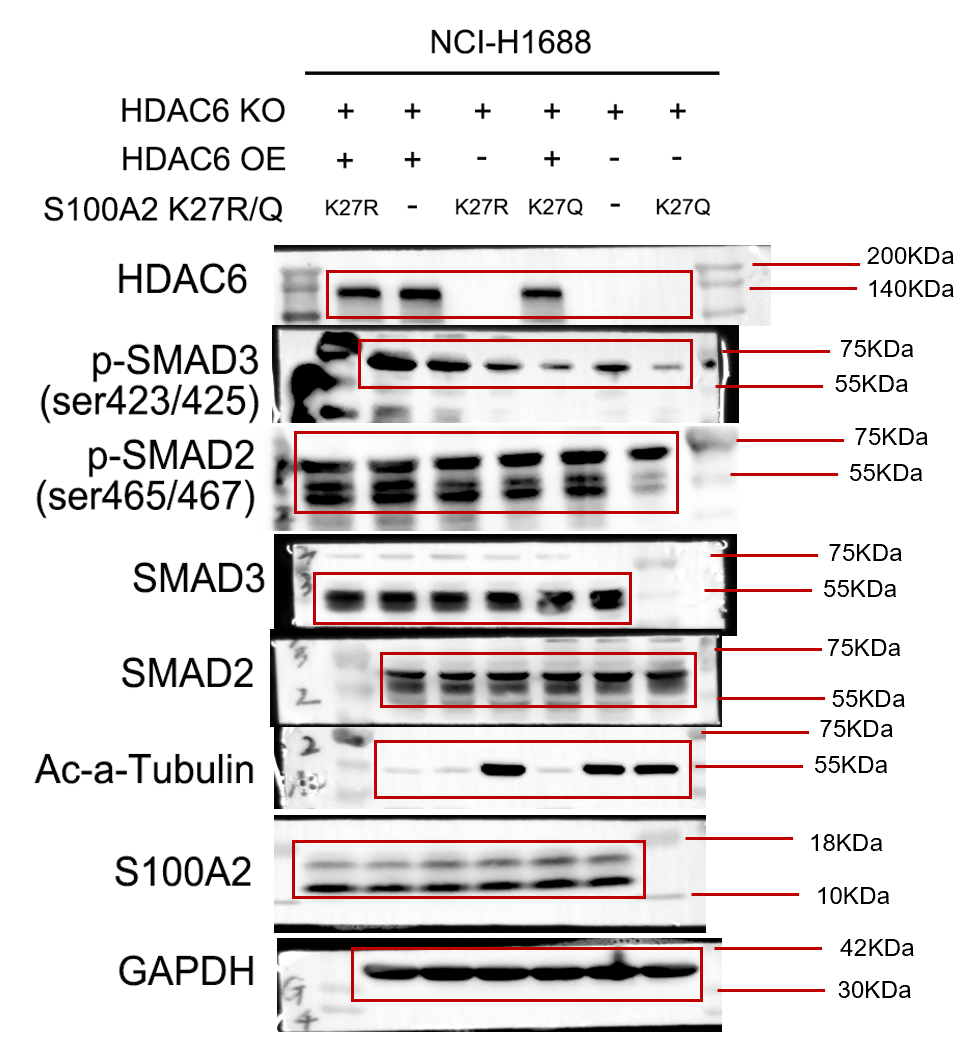
** **
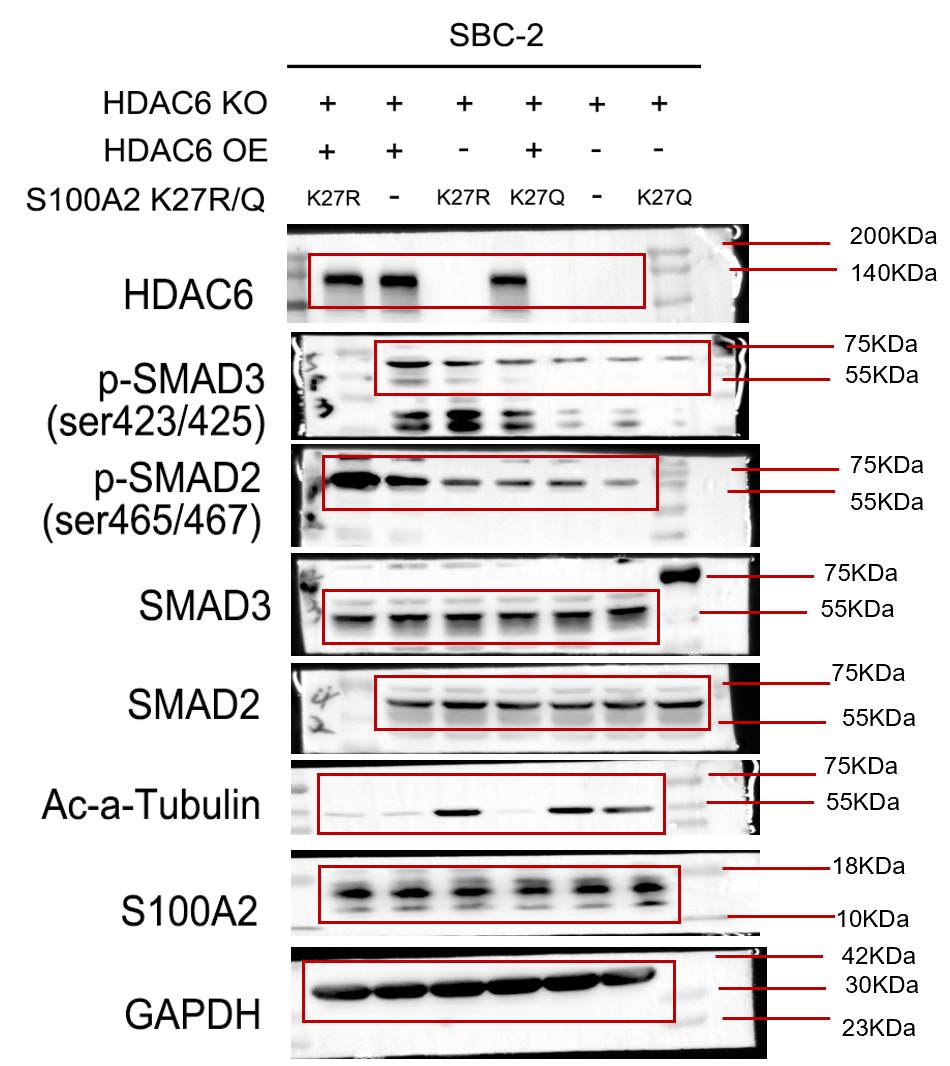
**

**Figure. 6g**

**
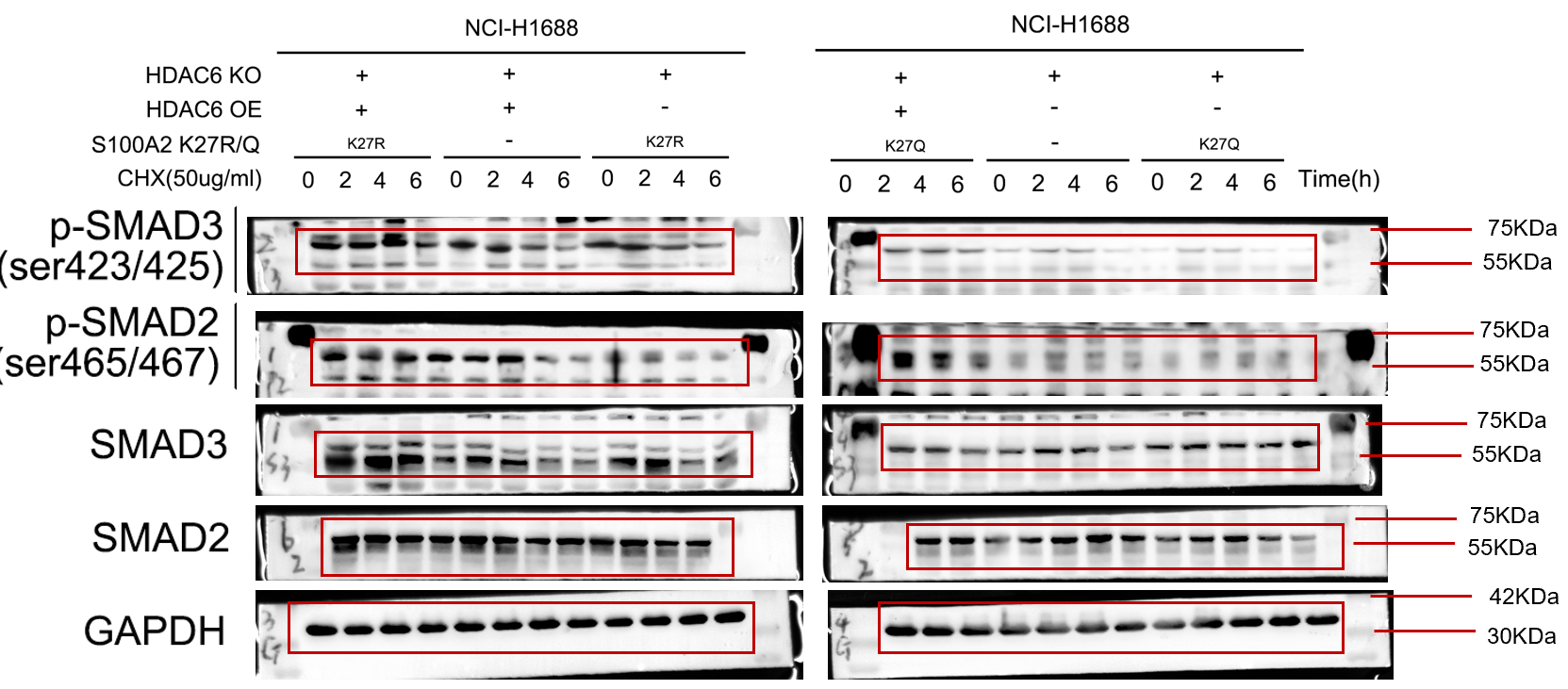
**

**Figure. 6h**

**
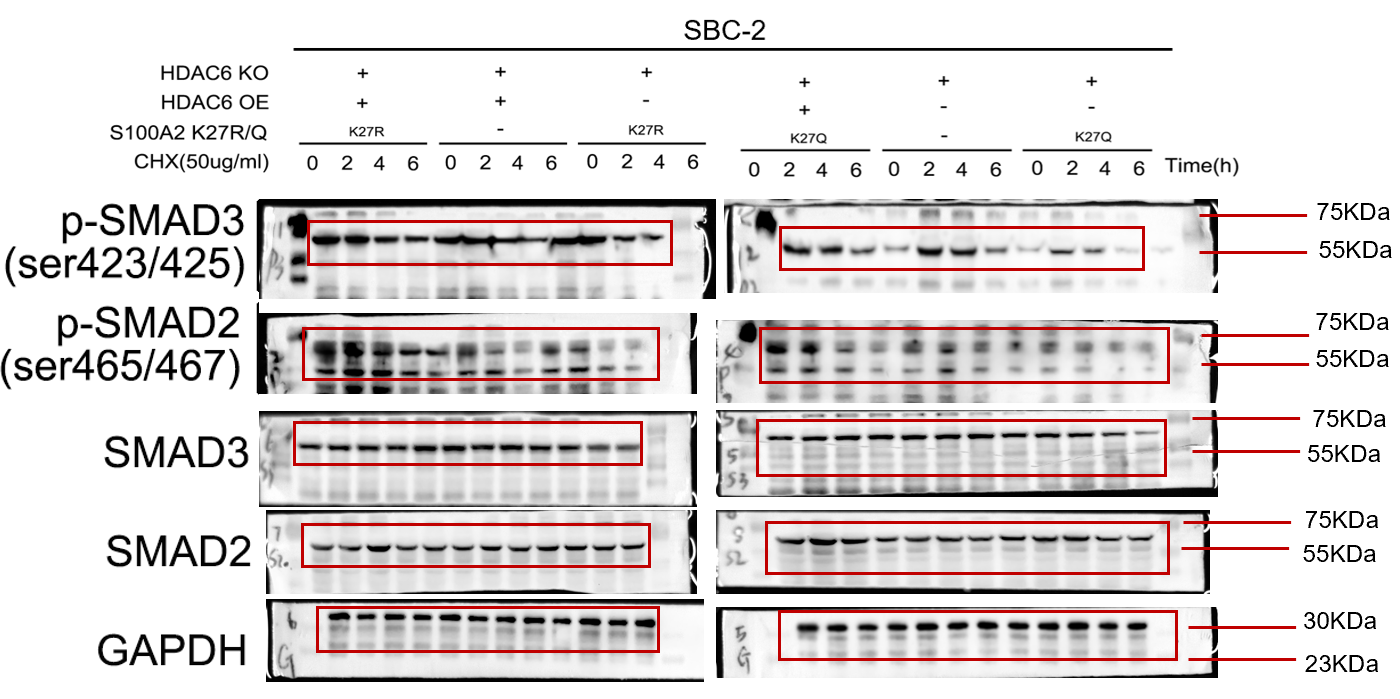
**

**Figure. 6i Figure. 6j**

**
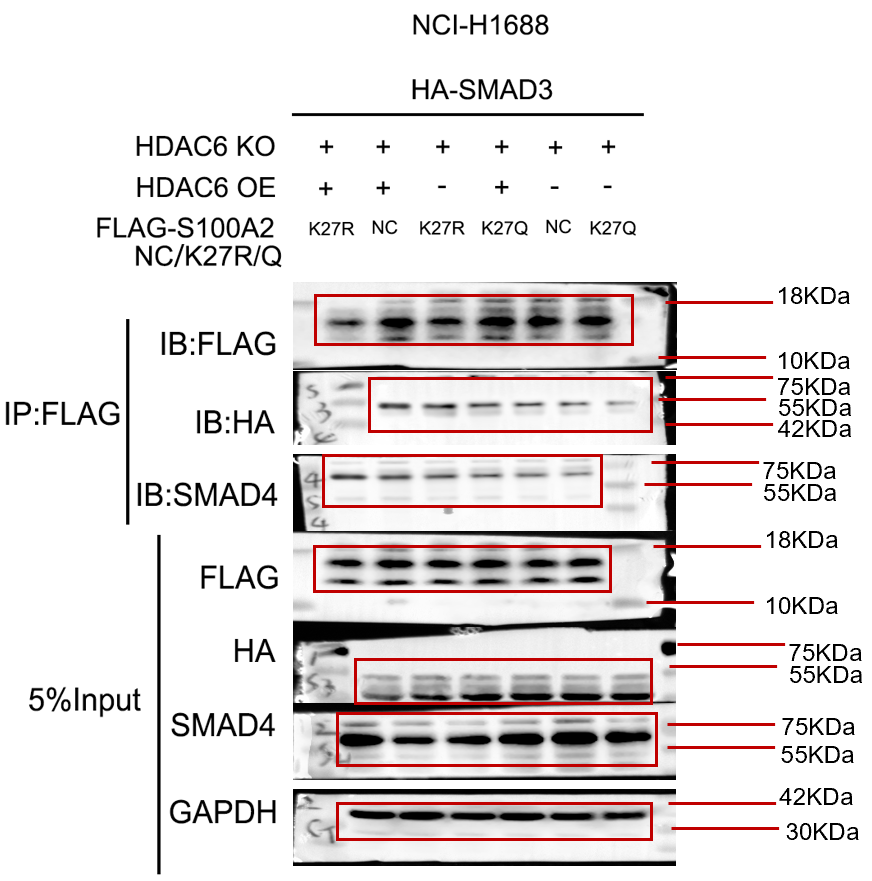
** **
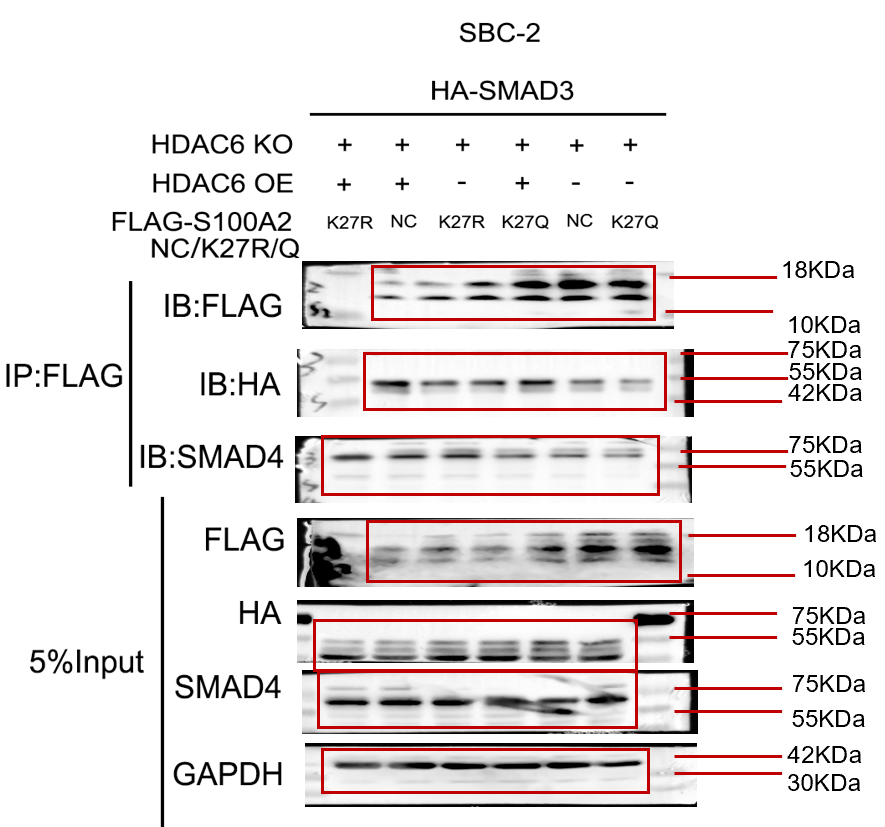
**

**Figure. 6k**

**
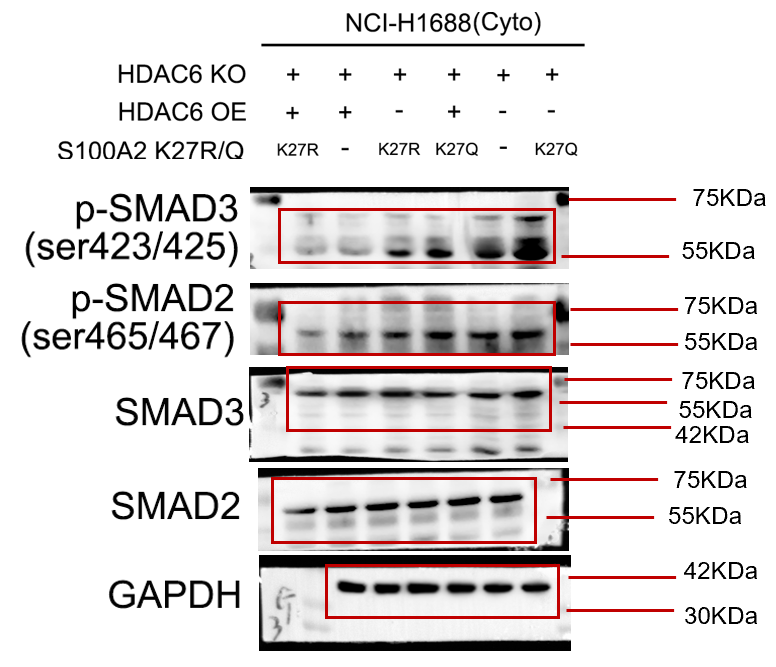

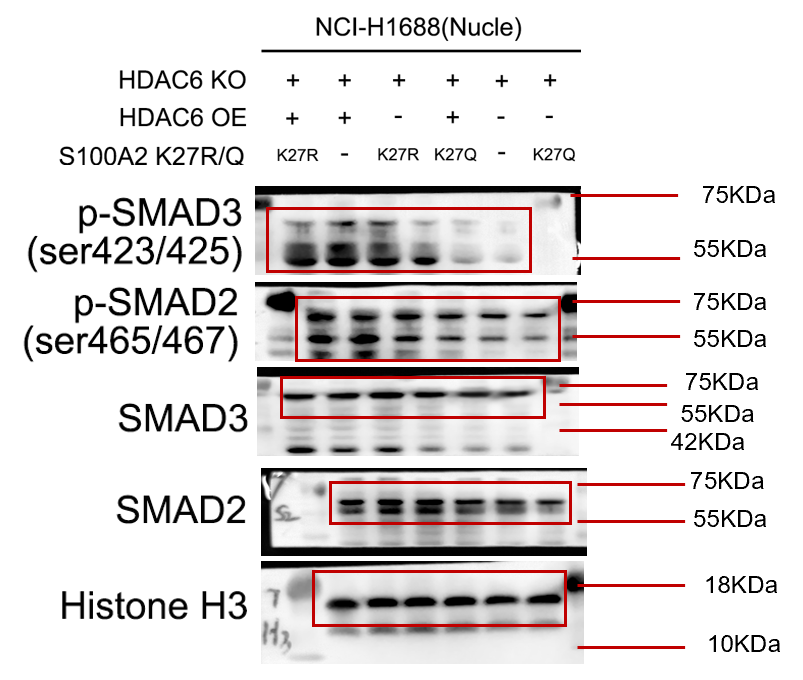
**

**Figure. 6l**

**
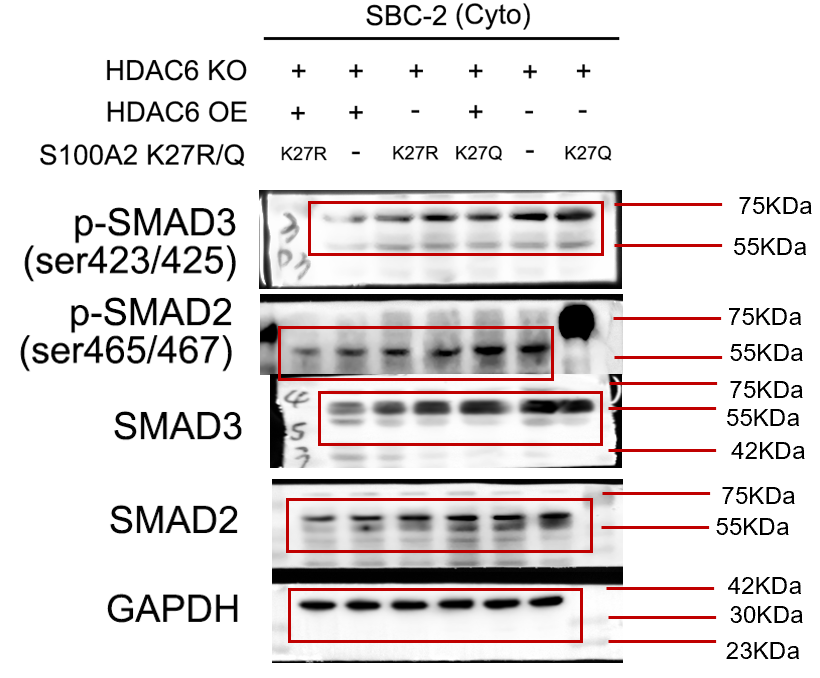

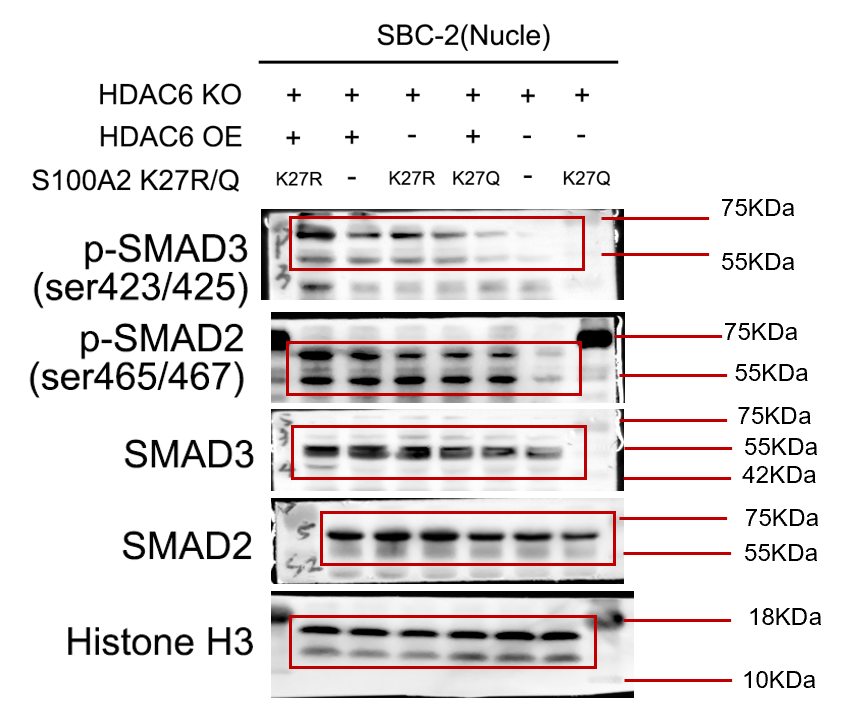
**

**Supplementary Figure. 1c Supplementary Figure. 1f**

**
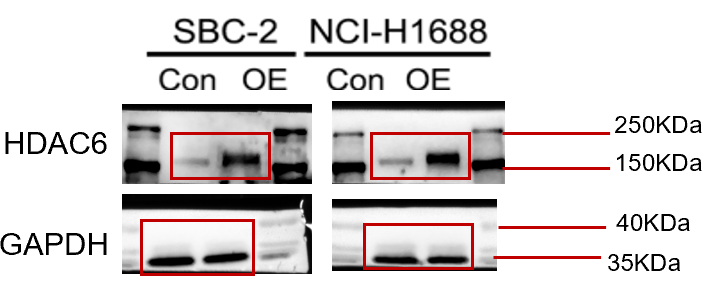
** **
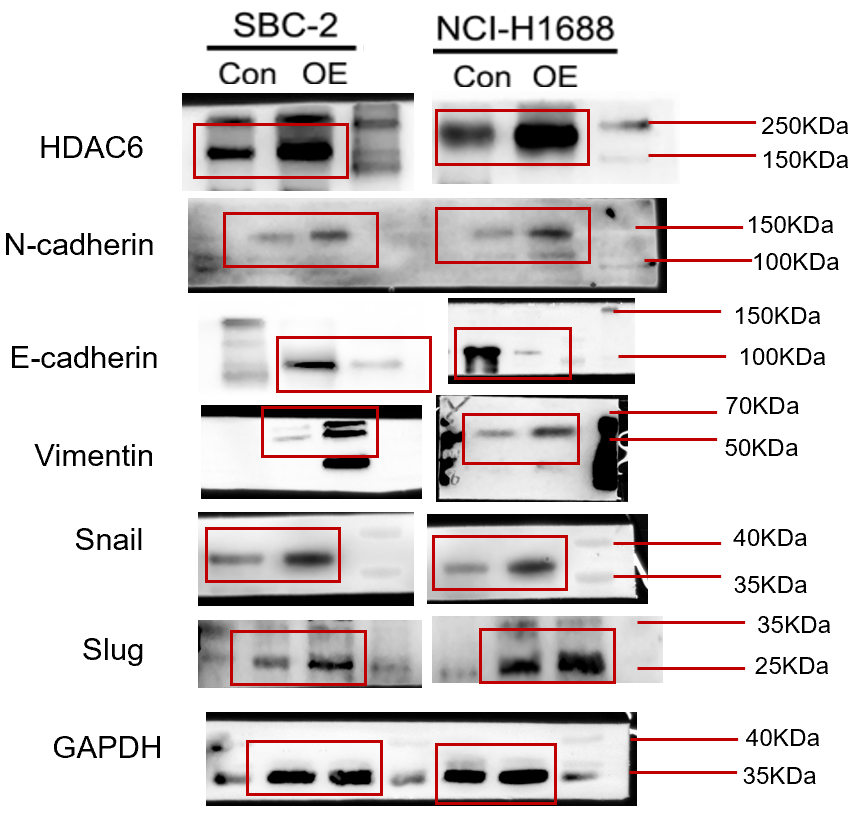
**

**Supplementary Figure. 1i Supplementary Figure. 2a**

**
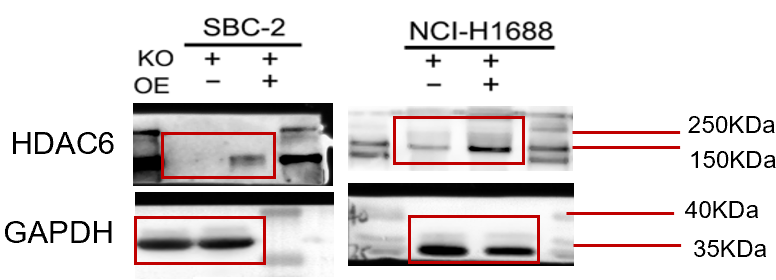

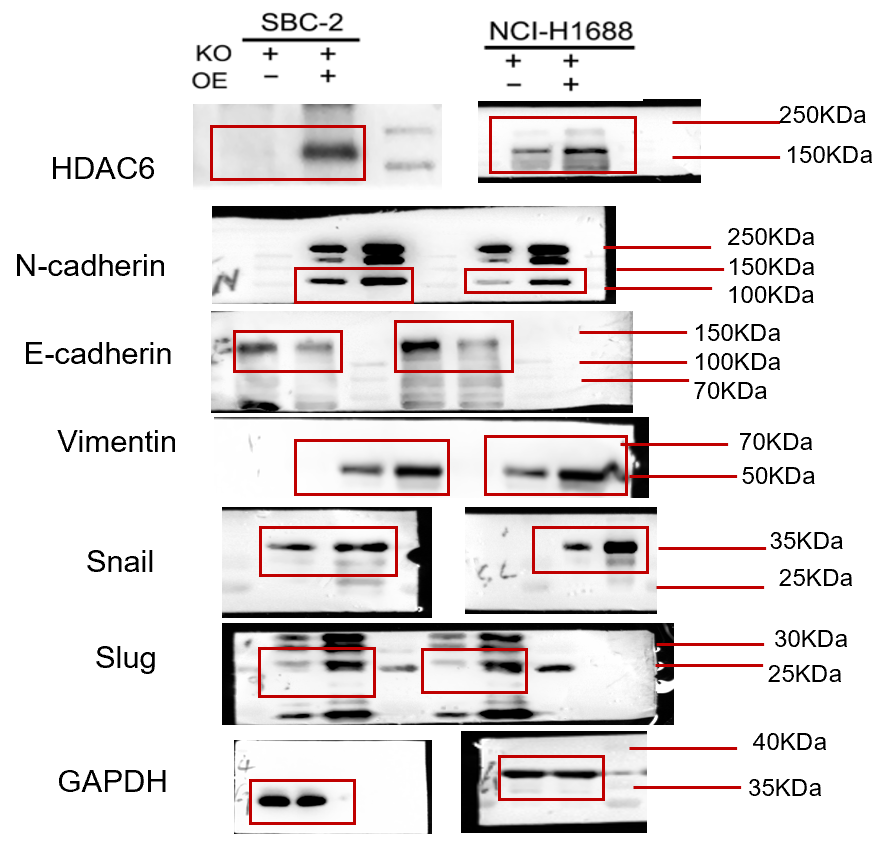
**

**Supplementary Figure. 2e and 2f Supplementary Figure. 2g

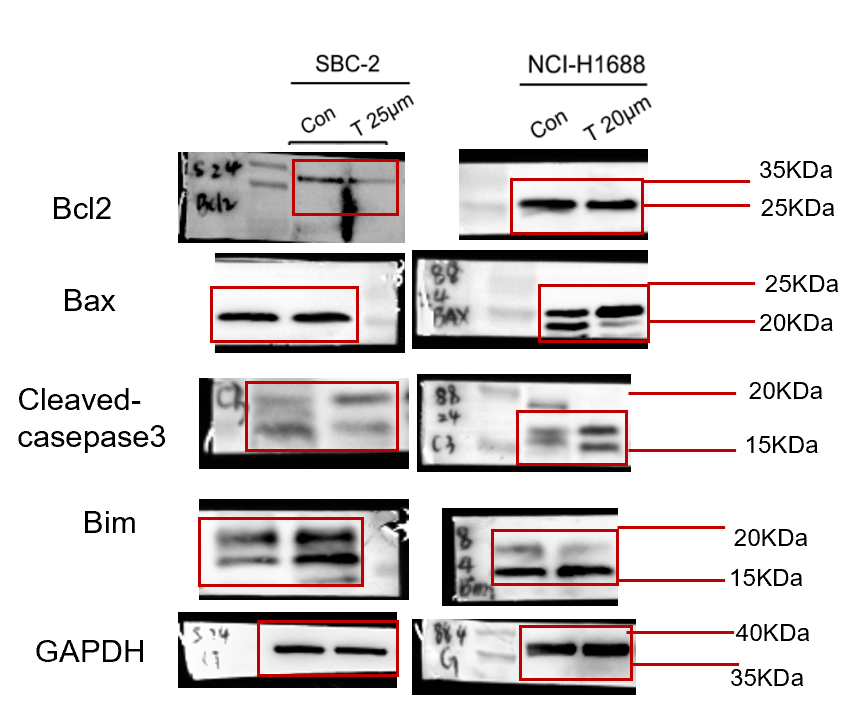
** **
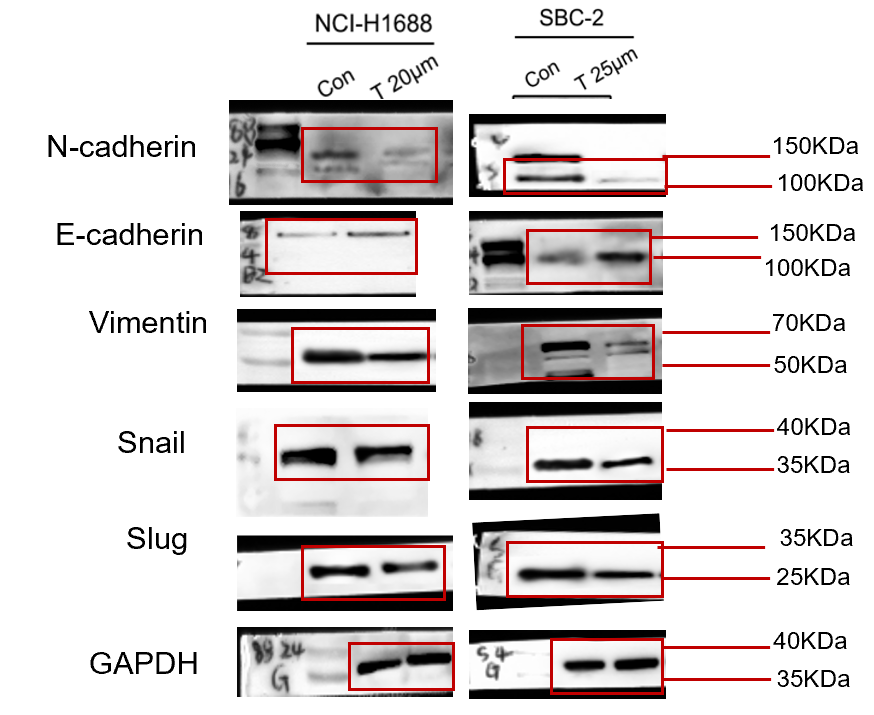
**

**Supplementary Figure. 2n**

**
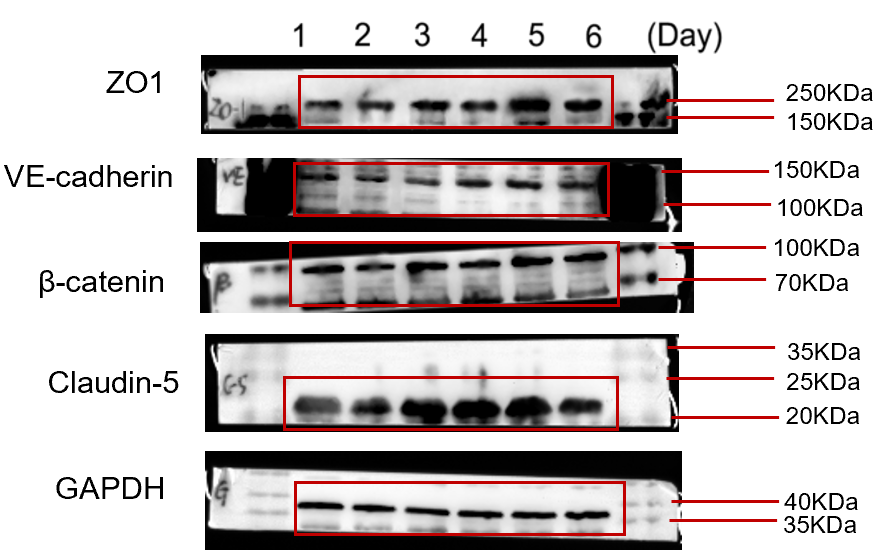
**

**Supplementary Figure. 2n Supplementary Figure. 2h**

**
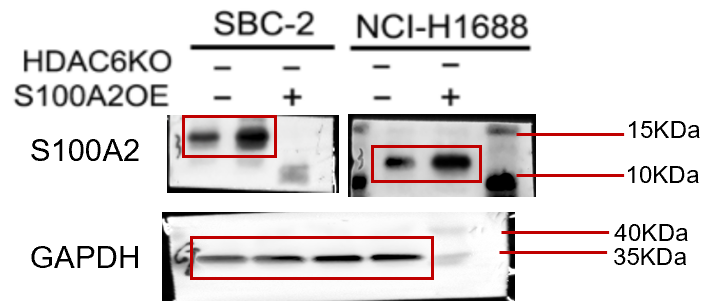
** **
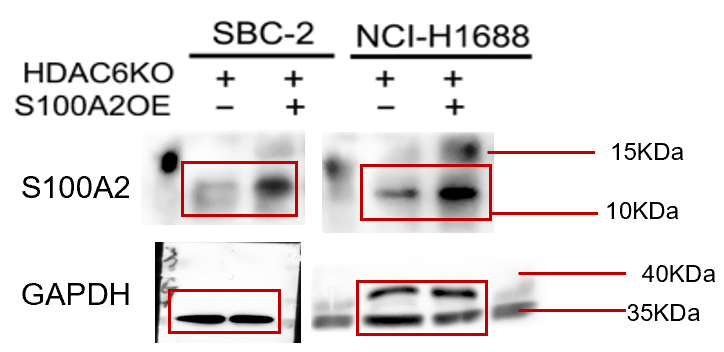
**

**Supplementary Figure. 5a and 5b**

**
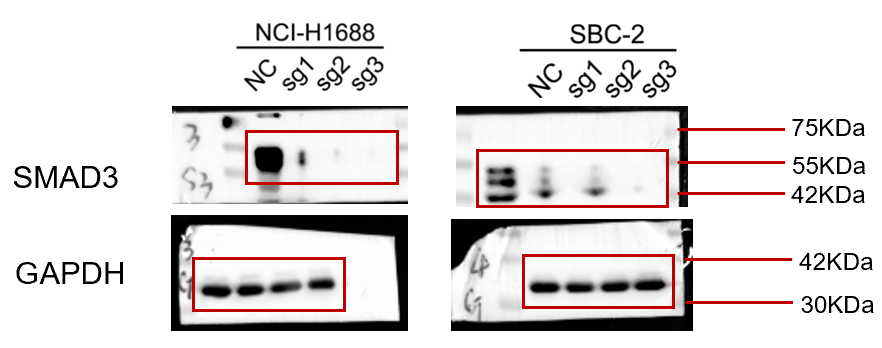
**

**Supplementary Figure. 5c**

**
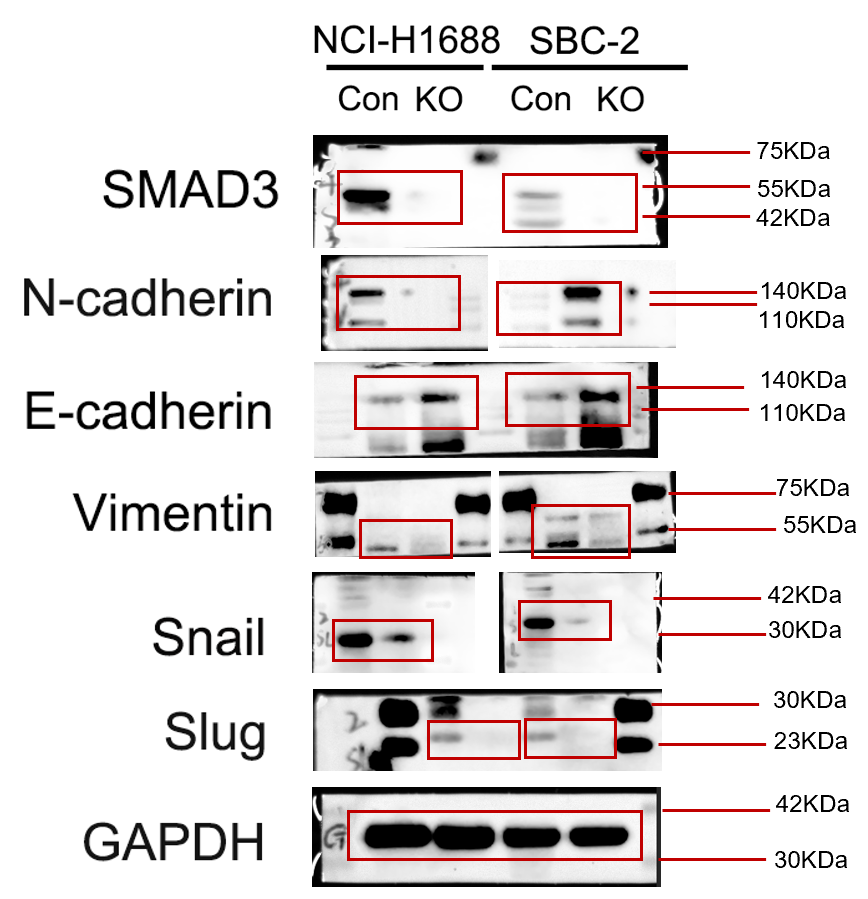
**
